# Supplementary figures and images for: Arktocara yakataga, a new fossil odontocete (Mammalia, Cetacea) from the Oligocene of Alaska and the antiquity of Platanistoidea
Source: PeerJ. 2016 Aug 16;4:e2321. doi: 10.7717/peerj.2321 (PMC4991871; doi:10.7717/peerj.2321)

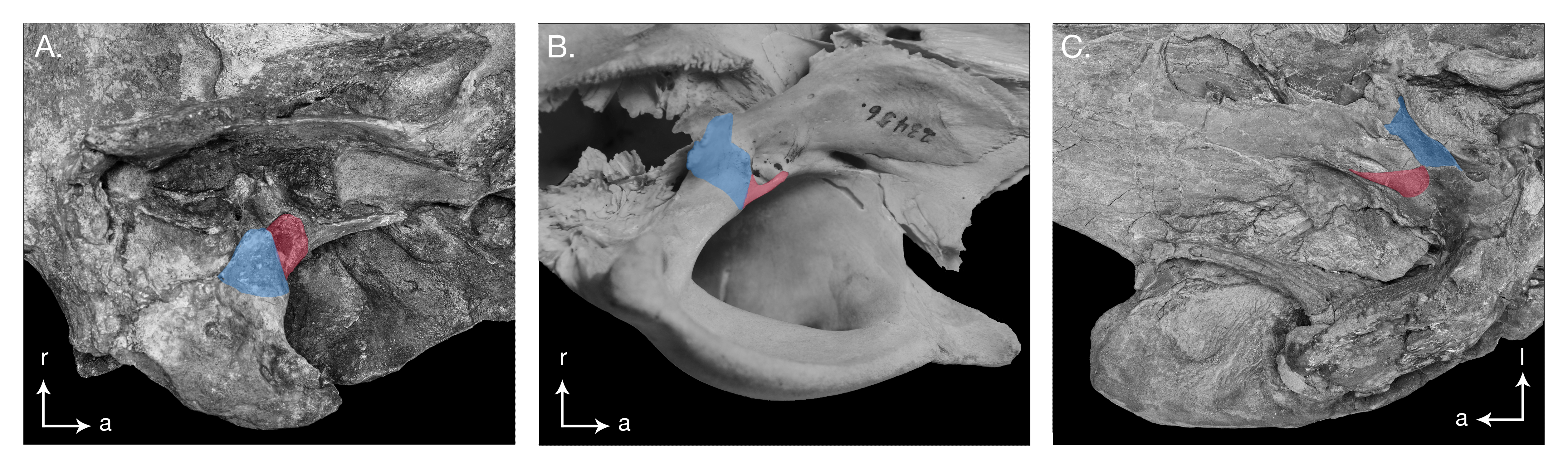

Supplement: Figure S1 — A homologous feature of the squamosal is observed in multiple platanistoid taxa: (A) Arktocara yakataga (USNM 214830), (B) Platanista gangetica (USNM 23456) and (C) USNM 214911, an undescribed platanistoid. This feature (highlighted in red), is a pointed lamina projected anteromedially from the anterior margin of the falciform process (highlighted in blue), posterior to the lateral lamina of the pterygoid and anterior to the posterolateral lamina of the alisphenoid that articulates with the base of the falciform process (see Figs. 3 and 6 for additional reference). Arrows indicate anatomical direction, with a, anterior, l, left lateral, and r, right lateral. [file peerj-04-2321-s001.png]

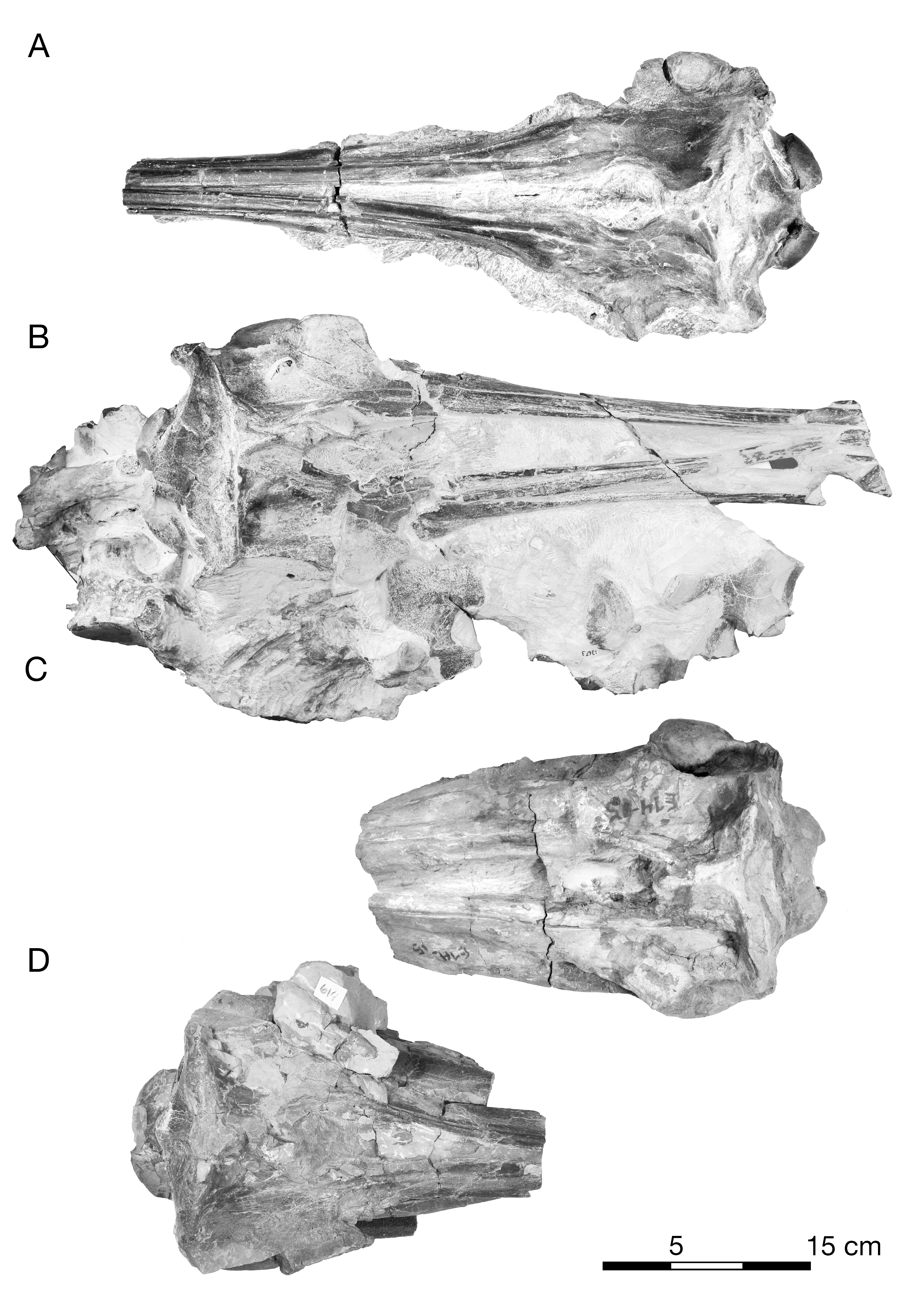

Supplement: Figure S2 — Photographs of undescribed platanistoid specimens housed in the Vertebrate Paleontology collections of the National Museum of Natural History, Smithsonian Institution, Washington D.C. All of the skulls are referred in this paper to the allodelphinid genus Goedertius. (A) USNM 335406, (B) USNM 335765, (C) USNM 314421, (D) USNM 13673. [file peerj-04-2321-s002.png]
