# Supplementary material for: Arktocara yakataga, a new fossil odontocete (Mammalia, Cetacea) from the Oligocene of Alaska and the antiquity of Platanistoidea
Source: PeerJ. 2016 Aug 16;4:e2321. doi: 10.7717/peerj.2321 (PMC4991871; doi:10.7717/peerj.2321)
Supplement: Appendix S1 — Following Tanaka & Fordyce (2015a). Modifications new to this study are noted. [file peerj-04-2321-s004.pdf]

S3 Appendix.

Morphological characters used in the phylogenetic analysis.

Terminology generally follows that of the cladistic papers cited, which in a few cases does not agree with the recommended uses of Mead and Fordyce (2009).

For each character, references are given for the main past uses, with the relevant published character number given with a hatch # thus: Murakami et al. (2012) #1.

We retain Tanaka & Fordyce (2015a)'s referencing formatting for ease of use.

An asterisk * indicates characters or states or state that were modified by Tanaka & Fordyce (2015a) from the version of the matrix in Tanaka & Fordyce (2014). See Appendix S4 in Tanaka & Fordyce (2015a) for further explanation.

An ~ indicates coding modifications by Boersma & Pyenson (2016). Modifications are listed at the end of the character state descriptions.

Rostrum, Dental, and Mandibular

(1) Length of rostrum as percent skull length: moderately long, 50–55% (0); long, 55–60% (1); very long, >60% (2); medium, 50–40% (3); very short, 40–35% (4). (Murakami et al. [2, 3] #1; modified from Arnold and Heinsohn, [4] #8; Bianucci, [5] #1; Lambert, [6] #1).

(2*) Premaxillae transverse proportion: transversely inflated almost entire length of rostrum (0); flat almost entire length of the rostrum (1). (Murakami et al. [2, 3] #2).

(3*) Premaxillae mediolateral proportion: not compressed mediolaterally (0); compressed mediolaterally at anterior of rostrum (1). (Murakami et al. [2, 3] #3).

(4) Premaxillae at apex of rostrum: with lateral margins parallel or diverging (0); narrowing (1). (Murakami et al. [2, 3] #4; modified from Bianucci, [5] #2).

(5) Maxilla length as percent rostrum length: short, <85%, tips of maxillae not reaching tip of rostrum, (0); long, >89%, tips of maxillae to within 10% of rostrum tip (1); same as state 1 except lack of alveoli (2). (Murakami et al. [2, 3] #5; modified from Lambert, [7] #1).

(6*) Mesorostral groove: V-shaped or U-shaped opening (0); partially or completely filled in with vomer, becoming a solid rod of bone (1); absent (2). (Messenger and McGuire, [8] #1429; Geisler and Sanders, [9]#5; Geisler et al., [10] [11] #5; Murakami et al. [2, 3] #6; derived from Moore, [12]).

(7*) Mesorostral groove constricted posteriorly, anterior to the nares and behind the level of the antorbital notch, then rapidly diverging anteriorly: absent (0); present (1). (modified from Murakami et al. [3] #279).

(8) Lateral margin of rostrum anterior to maxillary flange: concave (0); straight (1); convex (2); absent (3) (Murakami et al. [2, 3] #7; modified from Bianucci, [5] #3).

(9*) Rostral constriction: absent (0); constriction anterior to antorbital notch (1); constriction anterior to maxillary flange (2). (Murakami et al. [2, 3] #8; modified from Muizon, [13]; Barnes, [14]; Messenger and McGuire, [8] #1424; Geisler and Sanders, [9] #6; Geisler et al., [10, 11] #6).

(10) Antorbital notch: absent or weakly developed (0); well developed (1). (Messenger and McGuire, [8] #1426; Fajardo-Mellor et al., [15] #6; Murakami et al. [2, 3] #9).

(11) Width of premaxillae at mid-rostrum as percent greatest width of maxillae at level of postorbital processes: wide, >25% (0); medium, 25–15% (1); narrow, <15% (2). (Murakami et al. [2, 3] #10; modified from Aguirre-Fernandez et al., [16] #4) If the supraorbital process of the maxilla does not reach above the postorbital process, then the width is measured as a percent of the frontal width, excluding the postorbital process at the postorbital line.

(12) Width of rostrum at mid-length as percent greatest width of maxillae at level of postorbital processes: wide, >35% (0); medium, 35–30% (1); narrow, <30% (2). (Murakami et al. [2, 3] #11; modified from Aguirre-Fernandez et al., [16] #6).

(13) Width of rostrum at antorbital notch as percent greatest width of maxillae at level of postorbital processes: wide, >68% (0); medium, 68–45% (1); narrow, <45% (2). (Murakami et al. [2, 3] #12; modified from Geisler and Sanders, [9] #7; Geisler et al., [10, 11] #7).

(14) Premaxillae in dorsal view: contacting along midline for less than half length of rostrum (0); widely separated by mesorostral groove in rostrum (1); narrowly separated by mesorostral groove in rostrum (2); contacting along midline for approximately half the entire length or more than of rostrum but not fused (3); contacting along midline for approximately half the entire length or more than of rostrum and partially fused (4); converging (either contacting and separating) in mid-rostrum (5). (modified from Murakami et al. [2, 3] #13; modified from Muizon, [17]; Fordyce, [18] #52; Messenger and McGuire, [8] #1405; Geisler and Sanders, [9] (9); Geisler et al., [10] [11] #9).

(15) Suture between maxilla and premaxilla on rostrum: unfused except distal tip of rostrum

(0); fused partly or along most of rostrum (1). (Murakami et al. [2, 3] #14; modified from Fordyce, [18] #36; Messenger and McGuire #1418, [8]; Geisler and Sanders, [9] #10; Lambert, [7] #2; Geisler et al., [10] [11] #10).

(16*) Posterior wall of antorbital notch: maxilla (0); lacrimal and jugal, or maxilla appeared in small area posterior to antorbital notch parallel with lacrimal and jugal (1); no notch but horizontal groove inferred to be for the facial nerve in the maxilla laterally on the face well above the margin of the rostrum (2). (Murakami et al. [2, 3] #15; modified from Geisler and Sanders, [9] (15); Geisler et al., [10] [11] #15).

(17) Vomer anterior to maxilla-palatine suture in ventral view: not exposed (0); width of vomer <10% length of vomer, (1); vomer width ratio >10% vomer length (2). (Messenger and McGuire, [8] #1428; Murakami et al. [2, 3] #16; derived from Zhou [19].

(18) Anterior sinus fossa: absent (0); between anterior extremity of pterygoid sinus and posterior extremity of upper tooth row (1); between posterior extremity of upper tooth row and midpoint of rostrum (2); beyond midpoint of rostrum (3). (Murakami et al. [2, 3] #17; modified from Muizon, [17]; Barnes, [20]; Bianucci, [5] #13; Arnold and Heinsohn, [4] #21; Geisler and Sanders, [9] #157; Aguirre-Fernandez et al., [16] #18; Geisler et al., [10] [11] #157; derived from Fraser and Purves, [21]).

Teeth

(19) Number of double-rooted teeth in maxilla: 6–8 (0); 0 (1). (modified from Geisler and Sanders, [9] #23; Geisler et al., [10] [11] #23; Murakami et al. [2, 3] #18).

(20) Tooth enamel: reticulating striae (0); smooth (1); nodular (2); absent (3). (Murakami et al. [2, 3] #20; modified from Messenger and McGuire, [8] #1469; Geisler and Sanders, [9] #26; Geisler et al., [10, 11] #26; derived from Zhou, [19]).

(21*) Teeth: heterodont and some teeth with denticle (0); conical, with or without accessory cusp (1); spatulate (2); laterally compressed (3). (Murakami et al. [2, 3] #21; modified from Heyning, [22] #40, [23] #72; Arnold and Heinsohn,[4] #25; Messenger and McGuire, [8] #1470; Geisler and Sanders, [9] #27, [11] #27; Lambert, [6] #16; Geisler et al., [10] #27; derived from Barnes, [24]).

(22) Upper anterior "teeth": about same size as upper posterior teeth (0); greatly enlarged (1); clearly smaller than upper posterior teeth or absent (2). (modified from Murakami et al. [2, 3] #22).

(23) Buccal teeth ectocingulum: present (0); absent (1). (Murakami et al. [2, 3] #23; modified from Geisler and Sanders, [9] #31; Geisler et al., [10, 11] #31).

(24) Buccal teeth entocingulum: present (0); absent (1). (Geisler and Sanders, [9] #32; Geisler et al., [10, 11] #32; Murakami et al. [2, 3] #24).

(25) Greatest diameter of largest functional tooth as percent of greatest width of maxillae at the level of the postorbital processes: large, >5% (0); medium, 5–3% (1); small, <3% (2). (Murakami et al. [2, 3] #25; modified from Aguirre-Fernandez et al., [16] #15).

Mandible

(26) Anterior mandibular teeth: deeply rooted, root >50% of tooth (0); not deeply rooted, root <50% of tooth (1). (Messenger and McGuire, [8] #1471; Geisler and Sanders, [9] #28; Geisler et al., [10, 11] #28; Murakami et al. [2, 3] #26; derived from Flower, [25]; Moore, [12]).

(27*) Anterior-most mandibular "tooth": about same size as posterior teeth (0); smaller than posterior teeth (1); greatly enlarged (2); forming a tusk (3). (Murakami et al. [2, 3] #27; modified from Muizon, [26]; Geisler and Sanders, [9] #36; Messenger and McGuire, [8] #1477; Geisler et al., [10, 11] #36; derived from Flower, [25]).

(28) Number of teeth in mandible: 16–11 (0); 9–8 (1); 2 (2); 1 (3); 17–23 (4); 24–27 (5); 28–39 (6); >40 (7). (Murakami et al. [2] #28; modified from Messenger and McGuire, [8] #1468; Geisler and Sanders, [9] #37; Geisler et al., [10, 11] #37).

(29*) Length of mandibular symphysis as percent of mandible length: long, >20% (0); short, <20% (1). (Murakami et al. [2, 3] #29; modified from Messenger and McGuire, [8] #1465; Arnold and Heinsohn,[4] #7; Bianucci, [5] #26).

(30*) Mandibular symphysis: sutured but unfused (0); fused (1). (Fordyce, [18] #5; Messenger and McGuire, [8] #1466; Geisler and Sanders, [9] #40; Geisler et al., [10] [11] #40; Murakami et al. [2, 3] #30; derived from Flower, [27]).

(31*) Longitudinal groove on underside of mandibles: absent (0); present (1). (Geisler and Sanders, [9] #41; Geisler et al., [10, 11] #41; Murakami et al. [2, 3] #31; derived from Miller, [28]).

(32) Mandible: bowed medially (0); straight (1); slightly bowed laterally (2). (Sanders and Barnes, 2002; Geisler and Sanders, [9] #42; Geisler et al., [10] [11] #42; Murakami et al. [2, 3] #32; derived from Miller, [28]).

(33*) Elevation of coronoid process: very high (0); moderate (1); low (2). (Murakami et al. [2, 3] #33; modified from Geisler and Sanders, [9] #44; Bianucci, [5] #27; Geisler et al, [10, 11] #44).

Orbit

(34*) Antorbital process of maxilla in from dorsal view: triangular (0); robust and globose or rectilinear (1); absent (2). (Bianucci, [5] #4; Murakami et al. [2, 3] #34).

(35*) Angle of anterior edge of supraorbital process and the median line: oriented slightly anterolaterally, at an angle <30° (±) with sagittal plane (0) oriented anteromedially (1). (Murakami et al. [2, 3] #35; modified from Geisler and Sanders, [9] #49; Geisler et al., [10] [11] #49).

(36) Ratio of length of antorbital process of lacrimal to length of the orbit: <0.6 (0); ≥0.6 (1). (Murakami et al. [2, 3] #36).

(37) Lacrimal: wrapping around anterior edge of supraorbital process of frontal and slightly overlying its anterior end (0); appearing dorsally and forming most of antorbital process (1); appearing dorsally but not prominently in antorbital process (2); restricted to below the supraorbital process of maxilla (3). (Murakami et al. [2, 3] #37; modified from Geisler and Sanders, [9] #51; Geisler et al., [10, 11] #51; derived from Kellogg, [29]; Miller, [28]).

(38) Lacrimal foramen or groove: present (0); absent (1). (Geisler and Sanders, [9] #52; Geisler et al., [10, 11] #52; Murakami et al. [2, 3] #38).

(39*) Lacrimal and jugal: separated (0); fused (1). (Heyning, [22] #7, [23] #39; Geisler and Sanders, [9] #53; Geisler et al., [10, 11] #53; Murakami et al. [2, 3] #39; derived from Flower, [30]; Schulte, [31]; Miller, [28]).

(40*) Lacrimal and jugal: contacting each other externally (0); lacrimal excluded from edge of skull, jugal directly contacting anterior edge of frontal (1). (Geisler and Sanders, [9] #54; Geisler et al., [10, 11] #54; Murakami et al. [2, 3] #40; derived from Miller, [28]).

(41*) Jugal: thick and sturdy (0); thin, splint, or absent (1). (Geisler and Sanders, [9] #56; Lambert, [7] #21; Geisler et al., [10, 11] #56; Murakami et al. [2, 3] #41; derived from Miller, [28]; Barnes, [20]).

(42*) Combined anteroposterior length of the lacrimal and jugal exposure that is posterior to antorbital notch: With skull in ventral view, exposure is small and combined length forms <50% of anteroposterior distance from antorbital notch to postorbital ridge (0); intermediate, forms between 50 and 62% of that distance (1); large, forms between 62 and 69% that distance (2); very large, forms >69% of that distance (3). (Murakami et al. in [2, 3] #42; modified from Geisler and Sanders, [9] #55; Geisler et al., [10, 11] #55).

(43*) Dorsolateral edge of internal opening of infraorbital foramen: formed by maxilla (0); formed by maxilla and lacrimal and/or jugal (l); formed by lacrimal and/or jugal (2);

formed by frontal (3). (Geisler and Sanders, [9] #57; Geisler et al., [10] [11] #57; Murakami et al. [2, 3] #43; derived from Miller, [28]).

(44) Ventromedial edge of internal opening of infraorbital foramen: formed by maxilla (0); formed by maxilla and palatine and/or pterygoid (1); formed by palatine and/or pterygoid (2). (Geisler and Sanders, [9] #58; Geisler et al., [10, 11] #58; Murakami et al. [2, 3] #44; derived from Miller, [28]).

(45*) Maxillary tuberosity: present (0); absent (1). (Geisler and Sanders, [9] #59; Geisler et al., [10] #59, [11] #59; modified from Murakami et al. [2] #45; derived from Miller, [28]).

(46*) Direction of apex of postorbital process of frontal: projected posterolaterally and slightly ventrally (0); directed ventrally (1); not clear because of extremely reduced process (2). (modified from Murakami et al. [2, 3] #46; Geisler and Sanders, [9] #61; Geisler et al., [10, 11] #61).

(47*) Shape of postorbital process of frontal: robust, blunt descending posteriorly (0); pointed, attenuated, or acute triangular (1); triangular, trapezoidal, or an anteroposteriorly widened falciform (2); dorsoventrally long falciform (3). (modified from Murakami et al. [2, 3] #47).

(48) Frontal-maxilla suture angled posterodorsally at an angle of 50–70° (±) from axis of rostrum, with lateral exposure of frontal thickening posteriorly: absent (0); present (1). (Geisler and Sanders, [9] #48; Geisler et al., [10] #48, [11] #48; Murakami et al. [2, 3] #48; derived from Miller, [28]).

Facial Region

(49) Anterior dorsal infraorbital foramina: one (0); two (1); three or more (2). (Murakami et al. [2, 3] #49; modified from Barnes, [32]; Geisler and Sanders, [9] #64; Geisler et al., [10] #64, [11] #64).

(50) Rostral basin: absent or poorly defined (0); present, situated medial to antorbital notch and anterior to supraorbital process of frontal (1). (Geisler and Sanders, [9] #65; Geisler et al., [10] #65, [11] #65; Murakami et al. [2, 3] #50).

(51*) Width of premaxillae at antorbital notches as percent width of rostrum at antorbital notch: narrow, <49% (0); moderate, 50–64% (1); wide, >65% (2); antorbital notch absent (3). (Geisler and Sanders, [9] #66; Geisler et al., [10]#66, [11] #66; modified from Murakami et al. [2, 3] #51).

(52) Premaxillary foramina: absent (0); one on right side (1); two on right side (2); three on right side (3). (Geisler and Sanders, [9] #69; Geisler et al., [10] #69, [11] #69; Murakami

et al. [2, 3] #52; derived from Barnes, [20]).

(53*) Size of premaxillary foramen: right and left subequal (0); right much larger than left (1); premaxillary foramen absent (2). (Messenger and McGuire, [8] #1415; Murakami et al. [2, 3] #53; modified from Geisler and Sanders, [9] #70; Geisler et al., [10] #70, [11] #70).

(54*) Position of premaxillary foramen: anterior of antorbital notch and anterior edge of supraorbital process (0); approximately medial to or posterior to antorbital notch region (1); premaxillary foramen absent (2). (Geisler and Sanders, [9] #71; Geisler et al., [10] #71, [11] #71; Murakami et al. [2, 3] #54).

(55) Premaxillary foramen locating: medial (0); midpoint to lateral (1) absent (2). (modified from Murakami et al. [3] #280).

(56) Lateral margin of the right premaxilla posterior to premaxillary foramen: widen posteriorly (0); straight (1). (Murakami et al. [3] #281).

(57*) Posterolateral sulcus: deep (0); shallow or absent (1); presence of additional posterolateral sulcus (longitudinal striation) (2). (Murakami et al. [2, 3] #55; modified from Muizon, [13, 17]; Lambert, [6] #6; Geisler and Sanders, [9] #72; Geisler et al., [10] #72, [11] #72).

(58) Posterior projections of premaxillae: both premaxillae extending posterior to anterior tip of nasals (0); both premaxillae extending posterior to nasals (1); only right premaxillae extended posterior to nasal (2); neither premaxillae extending posterior to external nares, and narrow posterior end of premaxillae adjacent to external nares (3); neither premaxillae extending beyond external nares, and premaxillae displaced laterally by medial projection of maxilla (4); only right premaxilla extending beyond or in line with anterior-most portion of nasals (5). (Murakami et al. [2, 3] #76; modified from Muizon [13]; Barnes, [33]; Heyning, [22] #39, 42, [23] #63, 71, 74; Arnold and Heinsohn,[4] #35; Messenger and McGuire, [8] #1407, 1408; Fajardo-Mellor et al., [15] #3; Lambert, [6] #5; Fordyce, [18] #27).

(59*) Posterior dorsal infraorbital foramina of maxilla: absent (0); one (1); two or more (2); (Murakami et al. [2, 3] #57; modified from Barnes, [20]; Geisler and Sanders, [9] #75; Geisler et al., [10] #75, [11] #75).

(60) A posterior dorsal infraorbital foramen placed posteromedially, near posterior extremity of premaxilla: absent (0); present (1). (Fordyce, [18] #62; Lambert, [7] #13; Murakami et al. [2, 3] #58).

(61) Premaxillary sac fossae: absent (0); present (1). (Messenger and McGuire, [8] #1411; Lambert, [7] #4; Murakami et al. [2, 3] #59).

(62) Maxilla on dorsal surface of skull: does not contact supraoccipital posteriorly, maxilla separated by frontal and/or parietal (0); contact present (1). (Geisler and Sanders, [9] #129; Geisler et al., [10] #129, [11] #129, modified from Muizon, [26] [34]; Murakami et al., [2, 3] #60).

(63) Maxillae at anterior edge of supraorbital processes: abutting anterior edge of supraorbital processes of frontals (0); covering partially or almost completely surface of supraorbital processes (1). (Murakami et al. [2, 3] #61; modified from Fordyce, [18] #3; Messenger and McGuire, [8] #1419; Geisler and Sanders, [9] #76; Geisler et al., [10] #76, [11] #76; derived from Miller, [28]).

(64) Anterolateral corner of maxilla overlying supraorbital process of frontal: thin and equal in thickness to parts posteromedial (0); thickened with thinner maxilla in posteromedial direction (1). (Geisler and Sanders, [9] #78; Geisler et al., [10] #78, [11] #78; Murakami et al. [2, 3] #62).

(65) Pneumatic maxillary crest overhanging medially: absent (0); present (1). (Zhou, [19]; Heyning, [22] #26, [23] #58; Fordyce, [18] #66; Arnold and Heinsohn,[4] #14; Messenger and McGuire, [8] #1421; Murakami et al. [2, 3] #63).

(66*) Maxillary crest on supraorbital process of maxilla: longitudinal ridges absent except at lateral edge of antorbital process (0); presence of longitudinal ridge except at lateral edge of antorbital process (1); longitudinal ridge present and joined with maxillary flange (2); presence of transversely compressed high crest, except at lateral edge of antorbital process (3); absent (4). (Murakami et al. [2, 3] #64; modified from Muizon, [13] [35]; Barnes, [14]; Messenger and McGuire, [8] #1420; Geisler and Sanders, [9] #79; Geisler et al., [10] #79, [11] #79; derived from Miller, [28]).

(67) Anterior edge of nasals: anterior to, or in line with, anterior edges of supraorbital processes of frontals (0); posterior to anterior edges of supraorbital processes of frontals (1). (Murakami et al. [2, 3] #65; modified from Geisler and Sanders, [9] #80; Geisler et al., [10] #80, [11] #80).

(68*) Premaxillae in dorsal view: separated anterior to bony nares, exposing mesethmoid (0); joined premaxillae (or maxillae) closing at least posterior part of mesorostral groove (1). (Lambert, [7] #3; Murakami et al. [2, 3] #66).

(69*) Anterior edge of bony nares: inverted V-shaped, premaxillae gradually converging

anteriorly to midline (0); inverted U-shaped, premaxillae abruptly converging anteriorly to midline (1). (Muizon, [17]; Geisler and Sanders, [9] #81; Geisler et al., [10] #81, [11] #81; Murakami et al. [2, 3] #67).

(70) Fossa for inferior vestibule on maxilla lateral to external nares or lateral to premaxilla: absent (0); present (1). (Muizon, [17]; Murakami et al. [2, 3] #68; derived from Curry, [36]).

(71*) Maxillary intrusion, anterior to external nares and encroaching the posteromedial or medial face of each premaxilla: absent (0); maxilla visible within opened mesorostral canal as small exposure medially (1); exposure of maxilla reaches dorsally to level of premaxilla and forms a square, rectangular to triangular plate (2); exposure of maxilla reaches dorsally and forms a small subcircular to polygonal ossicle (3). (Muizon, [13] [17]; Arnold and Heinsohn,[4] #24; Messenger and McGuire, [8] #1422; Murakami et al. [2, 3] #69).

(72) Premaxillary crest or posterior maxillary crest adjacent to nasal: absent (0); present (1). (transverse premaxillary crest, sensu Lambert, [7] #6; Murakami et al. [2, 3] #70).

(73) Premaxilla: not overhanging itself or maxilla laterally (0); overhanging itself or maxilla laterally, from anterior to midpoint of external nares (1). (Murakami et al. [2, 3] #71).

(74) Premaxillary sac fossa: smooth (0); rugose (1). (Messenger and McGuire, [8] #1551; Murakami et al. [2, 3] #72).

(75) Ratio of width of right premaxilla to width of left premaxilla in line with midpoint of external nares: 0.90–1.19 (0); 1.20–1.50 (1); 1.50> (2). (Murakami et al. [2, 3] #73).

(76*) Ratio of greatest width of premaxillae to greatest width of maxillae at level of postorbital processes: ≥0.50 (0); 0.49–0.38 (1); <0.38 (2). (Murakami et al. [2, 3] #74).

(77*) Premaxillary eminence: absent (0); present but low (1); present and high (2). (Lambert, [6] #4; Murakami et al. [2, 3] #75; modified from Muizon, [13]; Barnes, [33]; Heyning, [22] #36, [23] #68; Arnold and Heinsohn,[4] #12; Messenger and McGuire, [8] #1410; Geisler and Sanders, [9]; #68; Fajardo-Mellor et al., [15] #2; Geisler et al., [10] #68, [11] #69; derived from Flower, [37]; Noble and Fraser, [38]).

(78) Right premaxilla: portion anterior to nasal opening wider than portion posterior to opening, with nasal septum angled anteriorly and to left (0); portion posterior to nasal opening wider than portion anterior to opening, with nasal septum angled anteriorly and to right (1). (modified from Geisler and Sanders, [9]; #86; Geisler et al., [10] #86, [11] #86; Murakami et al. [2, 3] #77).

(79*) Left bony naris: same size or slightly larger than right bony naris (0); at least twice the size of right bony naris (1). (Barnes, [20]; Geisler and Sanders, [9] #87; Geisler et al., [10] #87, [11] #87; Murakami et al. [2, 3] #78).

(80) Supracranial basin: absent (0); present (1). (Heyning, [22] #8, [23] #40; Fordyce, [18] #18; Messenger and McGuire, [8] #1400; Geisler and Sanders, [9] #88; Lambert, [7] #10; Geisler et al., [10] #88, [11] #88; Murakami et al. [2, 3] #79).

(81) Proximal ethmoid region: not visible in dorsal view, roofed over by nasals (0); exposed dorsally (1). (Messenger and McGuire, [8] #1455; Geisler and Sanders, [9] #92; Geisler et al., [10] #92, [11] #92; Murakami et al. [2, 3] #80; derived from Miller, [28]).

(82) Mesethmoid: not expanded posterodorsally (0); extended posterodorsally but narrow (1); expanded posterodorsally and visible in lateral view (2). (Murakami et al. [2, 3] #81; modified from Muizon, [13, 17]; Messenger and McGuire, [8] #1454; Bianucci, [5] #9).

Vertex and Area Adjacent to the Nares

(83) Inflections of ascending processes of premaxillae: gradual (0); vertical (1). (Geisler and Sanders, [9] #107; Geisler et al., [10] #107, [11] #107; modified from Murakami et al. [2, 3] #82; derived from Fordyce, [18]).

(84*) Inflections of ascending processes of premaxillae: gradual (0); abrupt (1). (Murakami et al. [2, 3] #83; modified from Geisler and Sanders, [9] #108; Geisler et al., [10] #108, [11] #108).

(85*) Premaxillary cleft: absent (0); present, posterior part of ascending processes of premaxillae bearing a distinct cleft, originating at posterior edge of premaxillae and continuing anteriorly, dividing each premaxilla into two (1); present, with shallow cleft (2). (Geisler and Sanders, [9] #109; Geisler et al., [10] #109, [11] #109; Murakami et al. [2, 3] #84).

(86) Nasal bones: two (0); one or zero (1). (Heyning, [22] #9, [23] #41; Murakami et al. [2, 3] #85; modified from Messenger and McGuire, [8] #1431; Geisler and Sanders, [9] #113; Geisler et al., [10] #113, [11] #113; derived from Kuzmin, [39]).

(87) Nasals: lower than frontals (0); nearly same height as frontals (1); clearly higher than frontals (2). (Muizon, [17]; Messenger and McGuire, [8] #1434; Geisler and Sanders, [9]; #124; Geisler et al., [10] #124, [11] #124; Murakami et al. [2] #86).

(88) Nasal protuberance: absent (0); present (1). (Muizon, [17]; Messenger and McGuire, [8] #1433; Fajardo-Mellor et al., [15] #7; Lambert, [6] #8; Murakami et al. [2, 3] #87).

(89) Both nasals: straight anterior edges in one transverse plane (0); with point on midline and gap on each side between premaxilla and nasal (1); concave posteriorly on midline and gap on each side between premaxilla and nasal (2); concave posteriorly on midline (3). (Murakami et al. [2, 3] #88; modified from Geisler and Sanders, [9] #116; Geisler et al., [10] #116, [11] #116; derived from Moore, [12]).

(90) Nasals: fossae on nasals absent (0); smooth-surfaced fossa on anterior to anterolateral surface (1). (Messenger and McGuire, [8] #1437; Murakami et al. [2, 3] #89).

(91) Transverse width of either of nasals as percent maximum length of nasals: very narrow, <20% (0); narrow, 21–69% (1); wide, >70% (2). (Murakami et al. [2] #90; modified from Muizon, [17]; Messenger and McGuire, [8] #1432; Geisler and Sanders, [9]; #119; Geisler et al., [10] #119, [11] #119).

(92) Nasals: medial portions roughly in same horizontal plane as, or higher than, lateral portions (0); medial portions depressed, forming a median trough immediately posterior to nasal openings (1). (Muizon, [17, 26]; Geisler and Sanders, [9]; #118; Geisler et al., [10] #118, [11] #118; Murakami et al. [2, 3] #91).

(93) Lateral edges of nasals: not overhanging or covering maxillae or premaxillae (0); overhanging or partly covering maxillae or premaxillae (1). (Murakami et al. [2, 3] #92).

(94*) Nasal-frontal suture: approximately straight transversely (0); anterior wedge (narial process) between frontal posterior ends of nasals (1); W or reversed U suture line (2). (Murakami et al. [2, 3] #93; modified from Muizon, [17]; Geisler and Sanders, [9] #121; Geisler et al., [10] #121, [11] #121).

(95) Frontals posterior to nasals and between premaxillae: wider than maximum transverse width across nasals (0); same as transverse width of nasals (1); narrower than transverse width of nasals, maxillae expanded medially posterior to nasals (2). (Geisler and Sanders, [9]#125; Geisler et al., [10] #125, [11] #125; Murakami et al. [2, 3] #94; modified from Messenger and McGuire, [8] #1457).

(96) Frontal boss on vertex: absent (0); present (1). (Muizon, [13, 17]; Messenger and McGuire, [8] #1461; Fajardo-Mellor et al., [15] #12; Murakami et al. [2, 3] #95; modified from Lambert, [6] #9).

(97*) Vertex: absent (0); present (1); highly developed (2). (Murakami et al. [2] #96; Muizon, [26]; Messenger and McGuire, [8] #1404; Lambert, [7] #7).

(98) Cranial vertex skewed asymmetrically to left side: absent (0); present (1). (Barnes, [20]; Bianucci, [5] #7; Aguirre-Fernandez et al., [16] #18; Murakami et al. [2, 3] #97).

(99) Anterodorsal wall of braincase: formed by frontals (0); mostly formed by maxillae (1). (Geisler and Sanders, [9] #127; Geisler et al., [10] #127, [11] #127; Murakami et al. [2, 3] #98; derived from Schulte, [31]; Miller, [28]).

(100) Nuchal crest: higher than frontals and/or nasals (0); at same level as frontals and/or nasals (1); below frontals and/or nasals (2). (Murakami et al. [2, 3] #99; modified from Geisler and Sanders, [9] #128; derived from Moore, [12]).

Temporal Fossae, Zygomatic Arch, and Occipitals

(101) Temporal fossa: not roofed over by lateral expansion of maxillae (0); roofed over by lateral expansion of maxillae (1). (Muizon, [17]; Heyning, [22] #22, [23] #54; Arnold and Heinsohn,[4] #39; Messenger and McGuire, [8] #1453; Murakami et al. [2, 3] #100).

(102) Roof of temporal fossa formed by: frontals (0); frontals, but with large opening through maxillae and/or premaxillae exposing margins of window formed by a frontal ring (1). (Geisler and Sanders, [9] #132; Geisler et al., [10] #132, [11] #132; Murakami et al. [2, 3] #101).

(103) Position and orientation of origin for temporal muscle on supraorbital process of frontal: origin laying on posterior face of supraorbital process and directed roughly posteriorly (0); origin lying on posteroventral face of supraorbital process and directed roughly ventrally (1). (Fordyce, [18] #8; Lambert, [7] #23; Murakami et al. [2, 3] #102).

(104*) Parietal dorsally: not fused to frontal or supraoccipital (0); completely fused to, and indistinguishable from, frontal or supraoccipital (1). (Murakami et al. [2, 3] #103).

(105) Parietals in dorsal view: contacting each other on the midline or separated by interparietal (0); in skull roof but visible only as small triangular areas at edges of intertemporal constriction, with supraoccipital overlapping and obscuring median portions (1); completely absent in skull roof (2); visible only as triangular areas, dorsolateral to supraoccipital, with non-overlapping supraoccipital separated from and contacting parietals along irregular suture (3). (Geisler and Sanders, [9] #134; Geisler et al., [10] #134, [11] #134; Murakami et al. [2, 3] #104; derived from Whitmore and Sanders, [40]; Barnes, [20]; modified from Lambert, [7] #15).

(106) Interparietal: present (0); absent or fused and therefore not distinguishable from parietals and frontals (1). (Geisler and Sanders, [9] #135; Geisler et al., [10] #135, [11] #135; Murakami et al. [2] #105).

(107) Sagittal crest for temporal muscle: present (0); absent (1). (Murakami et al. [2, 3] #106; modified from Geisler and Sanders, [9] #136; Geisler et al., [10] #136, [11] #136).

(108) Alisphenoid: broadly exposed laterally in temporal fossa (0); lateral surface broadly overlapped by parietal, with a narrow strip visible or invisible on ventral edge of temporal fossa in lateral view (1). (Geisler and Sanders, [9] #141; Geisler et al., [10] #141, [11] #141; Murakami et al. [2] #107).

(109*) Zygomatic process of squamosal: directed anterolaterally (0); directed anteriorly (1). (Sanders and Barnes, 2002; Geisler and Sanders, [9] #142; Geisler et al., [10] #142, [11] #142; Murakami et al. [2, 3] #108).

(110*) Zygomatic process of squamosal in lateral view: part of dorsal face visible (0); entire dorsal surface of squamosal visible (1). (Murakami et al. [2, 3] #109).

(111) Emargination of posterior edge of zygomatic process by sternomastoid muscle fossa, skull in lateral view: absent, posterior edge forming nearly right angle with dorsal edge of zygomatic process of squamosal (0); shallow emargination (1); deep emargination (2). (Geisler and Sanders, [9] #144; Geisler et al., [10] #144, [11] #144; Murakami et al. [2, 3] #110).

(112) Width of squamosal lateral to exoccipital as percent greatest width of exoccipitals, skull in posterior view: exposed portion of squamosal narrow, <15% (0); moderate, 16–35% (1). (modified from Geisler and Sanders, [9] #145; Geisler et al., [10] #145, [11] #145; Murakami et al. [2, 3] #111).

(113) Ventral edge of zygomatic process of squamosal in lateral view: concave (0); almost straight (1); convex (2). (Geisler and Sanders, [9]; #150; Geisler et al., [10] #150, [11] #150; Murakami et al. [2, 3] #112).

(114*) Postglenoid process of squamosal: not reduced (0); greatly reduced (1). (Murakami et al. [2, 3] #113).

(115*) Postglenoid process in lateral view: tapering ventrally (0); squared off ventrally (1); same as state 1 except very wide anteroposterior diameter of process (2). (Geisler and Sanders, [9] #151; Lambert, [7] #24; Geisler et al., [10] #151, [11] #151; Murakami et al. [2, 3] #114; derived from Muizon, [26]).

(116*) Relative ventral projections of postglenoid and post-tympanic processes of squamosal: postglenoid process more ventral or at same level as post-tympanic process (0); apex of postglenoid process dorsally higher than post-tympanic process (1). (Lambert, [7] #25; Murakami et al. [2]. b #115).

(117*) Nuchal crest in dorsoposterior view: semicircular, pointed anteriorly (0); rectangular or weakly convex anteriorly or posteriorly (1); convex posteriorly and/or midpoint convex

triangular and pointed anteriorly (2); prominently convex anteriorly (3); strongly convex posteriorly (4). (Murakami et al. [2, 3] #116; modified from Geisler and Sanders, [9]; #152; Geisler et al., [10] #152, [11] #152; derived from Barnes, [14]).

(118) Occipital shield: smoothly convex or concave (0); bearing distinct sagittal crest (1). (Sanders and Barnes, [41]; Geisler and Sanders, [9] #155; Geisler et al., [10] #155, [11] #155; Murakami et al. [2, 3] #117).

(119) Dorsal condyloid fossa: absent (0); present, situated anterodorsal to dorsal edge of condyle (1); present and forming deep pit (2). (Geisler and Sanders, [9] #156; Geisler et al., [10] #156, [11] #156; Murakami et al. [2, 3] #118; derived from Sanders and Barnes, [41]).

Anterior Basicranium

(120*) Palatine in nasal passage: thin, forming posterior part of nasal passage (0); thick, forming part of anterior wall of nasal cavities (1); palatine does not join anterior wall of nasal passage (2). (Murakami et al. [2, 3] #119; modified from Geisler and Sanders, [9]; #158; Geisler et al., [10] #158, [11] #158; derived from Miller, [28]).

(121*) Palatine exposure: exposed ventrally (0); partially covered by pterygoid, which divides it into medial and lateral exposures (1); ventral surfaces completely covered by pterygoids (2). (Muizon, [35]; Arnold and Heinsohn,[4] #15; Messenger and McGuire, [8] #1440; Geisler and Sanders, [9] #159; Lambert, [7] #27; Geisler et al., [10] #159, [11] #159; Murakami et al. [2, 3] #120; derived from Miller, [28]).

(122*) Lateral lamina of palatine: absent (0); present (1). (Muizon, [13, 17], [26]; Arnold and Heinsohn,[4] #16; Messenger and McGuire, [8] #1443; Murakami et al. [2, 3] #121).

(123) Lateral lamina of palatine: free from or sutured to maxilla (0); fused to maxilla (1). (Muizon, [17]; Messenger and McGuire, [8] #1439; Geisler and Sanders, [9] #161; Geisler et al., [10] #161, [11] #161; Murakami et al. [2, 3] #122).

(124*) Lateral lamina of palatine: does not form bony bridge “over” (= ventral to) orbit (0); does form bony bridge “over” (= ventral to) orbit (1). (Muizon, [13]; Messenger and McGuire, [8] #1444; Murakami et al. [2, 3] #123).

(125) Pterygoids in anteroventral view: separated from each other by posteroventrally elongated palatines and/or vomer (0); contacting entire length of hamular process (1); contacting each other partially (2). (Murakami et al. [2, 3] #124; modified from Arnold and Heinsohn,[4] #5; Messenger and McGuire, [8] #1445; Fajardo-Mellor et al., [15] #9; derived from Flower, [42]; Barnes, [33]; Marsh et al., [43]).

(126) Medial pterygoid-palatine suture in ventral view: angled anteromedially (0); nearly transverse (1); angled anterolaterally (2); angled anteroposteriorly (3). (Murakami et al. [2, 3] #125; modified from Geisler and Sanders, [9] #162; Geisler et al., [10] #162, [11] #162).

(127) Lateral lamina of pterygoid: absent (0); present and articulated with alisphenoid (1); partial, restricted to region lateral to hamular process (2). (Murakami et al. [2] #126; modified from Arnold and Heinsohn,[4] #121; Messenger and McGuire, [8] #1446; Geisler and Sanders, [9] #164; Lambert, [7] #32; Geisler et al., [10] #164, [11] #164; derived from Miller, [28]; Kellogg, [44]; Fraser and Purves, [21]).

(128) Subtemporal crest: present (0); present but reduced, or absent (1). (modified from Geisler and Sanders, [9] #165; Geisler et al., [10] #165, [11] #165; Murakami et al. [2, 3] #127).

(129) Superior lamina of pterygoid: absent from sphenoidal region but present in orbital region (0); present and covers most of ventral exposure of alisphenoid (1); partially absent from orbital region (2); completely absent from orbital region (3). (Murakami et al. [2, 3] #128; modified from Arnold and Heinsohn,[4] #16; Geisler and Sanders, [9] #167; Geisler et al., [10] #167, [11] #167; derived from Miller [28]; Fraser and Purves, [21]).

(130) Pterygoids excavated anterior to choanae by the pterygoid sinuses, with distinct anterior fossa clearly limited forwards by rounded wall: absent (0); present (1). (Lambert, [7] #28; Murakami et al. [2, 3] #129).

(131) Depth of pterygoid sinus fossa in basicranium: shallow or partially excavated (0); deep, excavated dorsally to level of cranial foramen oval (1); deep, and extended dorsally into orbit (2). (modified from Fordyce, [18] #6; Lambert, [7] #30; Murakami et al. [2, 3] #130).

(132) Anterior level of pterygoid sinus fossa: interrupted posterior to, or the level of, antorbital notch (0); extending beyond the level of the antorbital notch (1). (Lambert, [7] #29; Murakami et al. [2, 3] #131).

(133*) Preorbital and postorbital fossae of pterygoid sinuses: widened apices of preorbital and postorbital fossae of pterygoid sinuses present but fossae not merged or fused (0); widened apices of preorbital and postorbital fossae of pterygoid sinuses merged or fused dorsal to path of optic nerve (1). (Murakami et al. [2, 3] #132; modified from Muizon, [17]; Arnold and Heinsohn,[4] #19; Bianucci, [5] #10; Aguirre-Fernandez et al.,

[16] #19).

(134*) Fossa for preorbital lobe of pterygoid sinus in orbit: absent (0); present (1). (Fraser and Purves, [21]; Arnold and Heinsohn,[4] #18; Murakami et al. [2, 3] #133).

(135) Dorsal development of fossa for preorbital lobe of pterygoid sinus toward the frontal-maxilla suture: absent (0); present (1). (Muizon, [13, 17]; Heyning, [22] #37, [23] #69; Messenger and McGuire, [8] #1460; Arnold and Heinsohn,[4] #20; Lambert, [6] #13; Murakami et al. [2, 3] #134; modified from Fajardo-Mellor et al., [15] #13; derived from Fraser and Purves, [21]).

(136) Postorbital lobe of pterygoid sinus fossa: absent (0); present (1). (Arnold and Heinsohn,[4] #18; Geisler and Sanders, [9] #170; Geisler et al., [10] #170, [11] #170; Murakami et al. [2, 3] #135; derived from Fraser and Purves, [21]).

(137) Anteroposteriorly elongated pterygoid sinus fossa, at level of orbit, bordered by mediolaterally compressed subtemporal crest of frontal: absent (0); present (1). (Murakami et al. [2, 3] #136).

(138*) Orbitosphenoid: not contacting lacrimal or lacrimojugal (0); contacting lacrimal or lacrimojugal (1). (Murakami et al. [2, 3] #137).

(139*) Ratio of length of hamular process of pterygoid to cranium length: <0.30 (0); 0.30–0.44 (1); 0.45–0.59 (2); >0.60 (3). The length of the hamular process of the pterygoid is measured from anterior edge of the pterygoid to posterior edge of the hamular process. The cranium length is measured from anterior edge of the antorbital process to posterior edge of occipital condyles. (Murakami et al. [2, 3] #138; modified from Heyning, [22] #18, [23] #50; Muizon, [26]; Messenger and McGuire, [8] #1447; Lambert, [7] #31).

(140) Keel affecting ventral surfaces of hamular processes: absent (0); present (1). (Muizon, [17]; Messenger and McGuire, [8] #1449; Bianucci, [5] #14; Murakami et al. [2, 3] #139; modified from Fajardo-Mellor et al., [15] #10).

(141) Exposure of medial lamina of pterygoid hamuli in lateral view: complete or broad exposure due to extreme reduction of lateral lamina of pterygoid hamuli (0); no exposure due to a posterior extension of lateral lamina extending posterior to medial lamina (1); medial lamina of pterygoid hamuli exposing lateral lamina through ovoid window in lateral view (2). (Muizon, [17]; Fajardo-Mellor et al., [15] #11; Murakami et al. [2, 3] #140; derived from Noble and Fraser, [38]).

Posterior Basicranium

(142*) Falciform process of squamosal: plate-like with anteroposteriorly wide base (0); rod-like with narrow base (1); poorly developed or absent (2). (Geisler and Sanders, [9] #176; Geisler et al., [10] #176, [11] #176; Murakami et al. [2, 3] #141; modified from Lambert, [7] #36).

(143) Falciform process of squamosal: medial surface not sutured to lateral lamina of pterygoid (0); medial surface sutured to lateral lamina of pterygoid (1). (Murakami et al. [2, 3] #142; modified from Geisler and Sanders, [9] #177; Geisler et al., [10] #177, [11] #177).

(144*) Tympanosquamosal recess: absent, with anterior transverse ridge present (0); anterior transverse ridge absent and middle sinus inferred to be present without a large tympanosquamosal recess (1); present and enlarged, forming triangular fossa medial and anteromedial to postglenoid process (2); very large, forming large fossa bordering entire medial edge of glenoid fossa (3). (Geisler and Sanders, [9] #178; Geisler et al., [10] #178, [11] #178; Murakami et al. [2, 3] #143; modified from Lambert, [7] #35; derived from Fraser and Purves, [21], and Fordyce, [45]).

(145*) Bifurcation of tympanosquamosal recess: absent, almost undeveloped, elliptic (0); present, with a clear expansion anteriorly, invasion of mandibular fossa medially, and a depression (expansion) at the postglenoid process posteriorly (1). (Murakami et al. [2, 3] #144; modified from Muizon, [17]; Bianucci, [5] #11; Aguirre-Fernandez et al., [16] #20).

(146) Fossa for the basisphenoidal sinus: absent (0); present (1). (Fraser and Purves, [21]; Mead and Fordyce, [1]; Murakami et al. [2, 3] #145).

(147*) Position of more-distal part of alisphenoid-squamosal suture, with skull in ventral view: anterior to external opening of foramen oval or a homologous groove (0); courses along groove for mandibular branch of trigeminal nerve, or just posterior to it (1); just medial to anterior edge of floor of squamosal fossa, foramen ovale, and/or groove situated entirely on alisphenoid (2). (Geisler and Sanders, [9] #180; Geisler et al., [10] #180, [11] #180; Murakami et al. [2, 3] #146).

(148) Groove for mandibular branch of trigeminal nerve: lateral end of groove wrapping laterally around posterior end of pterygoid sinus fossa and opening primarily anteriorly (0); directed laterally and located entirely posterior to pterygoid sinus fossa (1). (Murakami et al. [2, 3] #147; modified from Geisler and Sanders, [9] #181; Geisler et al., [10] #181, [11] #181).

(149*) Suprameatal pit of squamosal: absent (0); present but shallow, situated dorsolateral to spiny process of squamosal (1); forming deep dorsolateral excavation into squamosal (2). (Geisler and Sanders, [9] #185; Geisler et al., [10] #185, [11] #185; Murakami et al. [2, 3] #149).

(150) Foramen spinosum: absent (0); present, located in anteromedial corner of anterior part of periotic fossa near or on squamosal-parietal suture (1). (Muizon, [34]; Geisler and Sanders, [9]#186; Geisler et al., [10] #186, [11] #186; Murakami et al. [2, 3] #150).

(151*) Posterior portion of periotic fossa of squamosal: fossa absent (0); fossa present but shallow (1); highly compressed fossa forming narrow slit or small blind foramen (2); posteromedial portion contains large deep fossa (3). (Geisler and Sanders, [9] #187; Geisler et al., [10] #187, [11] #187; Murakami et al. [2, 3] #149 and #151).

(152) Length of zygomatic process of squamosal as percent of greatest width of maxilla at postorbital process: >31% (0); ≤30% (1). (Murakami et al. [2, 3] #152; modified from Heyning, [22] #33, 35, #65, 67; Geisler and Sanders, [9] #188; Geisler et al., [10] #188, [11] #188) If the maxilla does not reach above the postorbital process, then use the frontal above the postorbital process.

(153) External auditory meatus: wide (0); narrow (1). (Fordyce, [18] #10; Geisler and Sanders, [9] #189, [11] #189; Lambert, [7] #26; Geisler et al., [10] #189; Murakami et al. [2, 3] #153).

(154*) Vomer: posterior edge terminating on or at anterior edge of basisphenoid (0); terminating on basioccipital, covering basioccipital-basisphenoid suture ventrally (1). (Barnes, [32]; Geisler and Sanders, [9] #190; Geisler et al., [10] #190, [11] #190; Murakami et al. [2, 3] #154).

(155) Rectus capitus anticus muscle fossa: absent or poorly developed (0); present with well-defined anterior edge (1). (Geisler and Sanders, [9] #192; Geisler et al., [10] #192, [11] #192; Murakami et al. [2, 3] #155).

(156*) Posteroventral-most point of basioccipital crest: rounded over (0); forming closely appressed separate flange, with narrow crease separating it dorsally from rest of basioccipital crest (1); projecting distinct flange posteriorly (2); distinct but separated by pronounced notch, interrupting basioccipital crest (3). (Geisler and Sanders, [9] #193; Geisler et al., [10] #193, [11] #193; Murakami et al. [2, 3] #156).

(157*) Angle formed by basioccipital crests in ventral view: parallel with no angle formed (0); ca. 15–40° (1); ca. 42–68° (2); ca. 70–90° (3); >100° (4). (Murakami et al. [2, 3] #157;

modified from Geisler and Sanders, [9] #194; Geisler et al., [10] #194, [11] #194).

(158*) Hypoglossal foramen: separated from jugular foramen, or jugular notch, by thick bone (0); separated by very thin bone or absent, in latter case hypoglossal foramen becoming confluent with jugular foramen (1). (Geisler and Sanders, [9] #195; Geisler et al., [10] #195, [11] #195; Murakami et al. [2, 3] #158).

(159*) Jugular notch, gap between paroccipital process and basioccipital crest: open notch, width of opening and depth of notch about equal (0); narrow and almost slit-like, depth much greater than width of opening (1). (Geisler and Sanders, [9] #196; Geisler et al., [10] #196, [11] #196; Murakami et al. [2, 3] #159).

(160) Paroccipital process, skull in ventral view: posterior edge located well anterior to the posterior edge of condyle (0); posterior edge in transverse line with posterior edge of condyle (1). (Geisler and Sanders, [9] #197; Geisler et al., [10] #197, [11] #197; Murakami et al. [2, 3] #160).

(161) Fossa for posterior sinus in exoccipital: absent or slightly concave (0); moderately concave (1); forming deep sack-like structure (2). (Murakami et al. [2, 3] #161; modified from Muizon, [26]; Lambert, [7] #38).

Malleus

(162) Tuberculum of malleus: unreduced (0); highly reduced, almost indistinguishable from articular head (1). (Muizon, [46]; Messenger and McGuire, [8] #1499; Geisler and Sanders, [9] #198; Geisler et al., [10] #198, [11] #198; Murakami et al. [2, 3] #162; modified from Lambert, [7] #69. derived from Doran, [47]).

(163) Processus muscularis of malleus: shorter than manubrium of malleus (0); sub-equal or longer than manubrium (1). (Murakami et al. [2, 3] #163; modified from Muizon, [17, 46]; Messenger and McGuire, [8] #1550; Geisler and Sanders, [9] #199; Lambert, [7] #70; Geisler et al., [10] #199, [11] #199).

Periotic

(164) Length of anterior process of periotic as percent length of pars cochlearis: short, <59% (0); long, >60% (1). (Murakami et al. [2, 3] #164; modified from Muizon, [17]; Heyning, [22] #5; Messenger and McGuire, [8] #1489; Geisler and Luo, [48] #1; Luo and Marsh, [49] #24; Geisler and Sanders, [9] #203; Lambert, [7] #39; Geisler et al., [10] #203, [11] #203; derived from Kellogg, [44]; Yamada, [50]; Kasuya, [51]).

(165) Apex of anterior process of periotic in dorsal view: pointed (0); dorsal edge of anterior process showing highly rounded or oblique edge due to its reduction with or without

pointed apex (1); thickened by prominent dorsal tubercle giving apex rectangular section in plane of body of periotic (2). (Murakami et al. [2, 3] #165; modified from Fordyce, [18] #53; Lambert, [7] #40).

(166) Lateral groove or depression affecting profile of periotic as viewed dorsally: no obvious vertical groove dorsal to hiatus epitympanicus (0); groove present with overall profile of periotic becoming slightly to markedly sigmoidal in dorsal view (1). (Fordyce, [18] #35; Murakami et al. [2, 3] #166).

(167) Anteroposterior ridge on dorsal side: undeveloped (0); developed on anterior process and body of periotic, associated with development of depression adjacent to groove for tensor tympani (1). (Fordyce, [18] #55; Murakami et al. [2, 3] #167).

(168*) Articulation of anterior process of periotic to outer lip of tympanic bulla: contact of ventral surface of anterior process of periotic with outer lip of tympanic bulla (0); contact with thickened rim of outer lip of tympanic bulla and additionally with accessory ossicle (1); contact only with accessory ossicle (2). (Luo and Marsh, [49] #7; Lambert, [7] #46; Murakami et al. [2, 3] #168).

(169*) Parabullary sulcus: absent (0); strongly curved, C-shape (1); weakly curved (2); strongly curved, V-shape (3). (modified from Fordyce, [18] #56 Anteroexternal sulcus).

(170*) Parabullary ridge of periotic: absent (0); present (1); present with a fossa between anterior process and parabullary ridge (2). (modified from Murakami et al. [2, 3] #171; Bianucci, [5] #15).

(171*) Articulation of anterior process with squamosal: extensive, most of lateral side contacting squamosal (0); large centrally-oriented ovoid region contacting squamosal, free around edges (1); small area of contact with squamosal (2); contact absent, articulation via ligaments (3). (Geisler and Sanders, [9] #207; Geisler et al., [10] #207, [11] #207; Murakami et al. [2, 3] #172; modified from Heyning, [23] #32; Messenger and McGuire, [8] #1490; derived from Heyning, [22]).

(172*) Anterior bullar facet: present (0); absent (1). (Muizon, [13, 17, 26]; Messenger and McGuire, [8] #1496; Lambert, [7] #42; Murakami et al. [2, 3] #173; modified from Fordyce, [18] #4; derived from Kellogg, [44]).

(173*) Anterior incisure: deep, pocket-like fossa with anterior groove (0); anterior groove only (1). (Geisler and Luo, [48] #7; Luo and Marsh, [49] #15; Geisler and Sanders, [9] #217; Geisler et al., [10] #217, [11] #217; Murakami et al. [2, 3] #174).

(174*) Fenestra rotunda: oval to subrounded (0); shaped like teardrop with fissure directed

toward aperture for cochlear aqueduct (1). (Fordyce, [18] #22; Geisler and Sanders, [9] #222; Lambert, [7] #49; Geisler et al., [10] #222, [11] #222; Murakami et al. [2, 3] #175).

(175*) Dorsal surface of periotic in lateral view: convex dorsally (0); pyramidal process convex dorsally (1); nearly flat (2). (Murakami et al. [2, 3] #176; modified from Luo and Marsh, [49] #18).

(176*) Relative position of dorsal depth of stapedial muscle fossa and fenestra rotunda: ventral to, or in line with, dorsal edge of fenestra rotunda (0); well dorsal to fenestra rotunda (1). (Geisler and Sanders, [9] #223; Geisler et al., [10] #223, [11] #223; Murakami et al. [2, 3] #177).

(177*) Posterodorsal edge of stapedial muscle fossa: absent, rounded lip (0); present (1). (Geisler and Luo, [48] #14; Geisler and Sanders, [9] #217; Geisler et al., [10] #217, [11] #217; Murakami et al. [2, 3] #178).

(178*) Caudal tympanic process of periotic: low, its ventral and posterior edges drawing smooth curve (0); Elevated, its ventral and posterior edges forming a right angle in medial view (1). (Geisler and Sanders, [9] #225; Geisler et al., [10] #225, [11] #225; Murakami et al. [2, 3] #179).

(179) Position of aperture for cochlear aqueduct: dorsomedial (0); medial (1). (Lambert, [7] #51; Murakami et al. [2, 3] #180).

(180) Aperture for cochlear aqueduct: smaller than aperture for vestibular aqueduct (0); approximately same size as aperture for vestibular aqueduct (1); much larger than aperture for vestibular aqueduct, with narrow posterior edge (2). (Geisler and Sanders, [9] #227; Geisler et al., [10] #227, [11] #227; Murakami et al. [2, 3] #181; modified from Muizon, [35]; Fordyce, [18]; Lambert, [7] #52).

(181) Excavation of tegmen tympani at base of anterior process: absent (0); present, with fossa on dorsolateral side of tegmen tympani (1). (Geisler and Sanders, [9] #231; Geisler et al., [10] #231, [11] #231; Murakami et al. [2, 3] #182).

(182*) Fundus of internal acoustic meatus: funnel-like, smaller at blind end and wider near rim (0); tubular (1). (Luo and Marsh, [49] #31; Geisler and Sanders, [9] #234; Geisler et al., [10] #234, [11] #234; Murakami et al. [2, 3] #183).

(183*) Internal acoustic meatus: pyriform (0); circular (1). (Muizon, [13]; Messenger and McGuire, [8] #1498; Bianucci, [5] #21; Murakami et al. [2, 3] #184).

(184) Lateral wall of internal acoustic meatus: high, with wedge-shaped area of elevated bone occurring between dorsal edge of tegmen tympani and internal acoustic meatus, the latter extending ventrally and increasing its depth (0); low, not protruding noticeably from fossa and surrounding bone (1). (Murakami et al. [2, 3] #185; modified from Geisler and Sanders, [9] #235; Geisler et al., [10] #235, [11] #235).

(185*) Aperture for vestibular aqueduct, in dorsal view: at transverse level of spiral cribriform tract (0); more lateral than spiral cribriform tract (1) (Lambert, [7] #53; Murakami et al. [2, 3] #186).

(186*) Articular rim: absent (0); present but small, forming ridge anterolateral to articulation surface of posterior process of periotic and separated from it by sulcus (1); present, sigmoidal and laterally elongated with hook-like process (2). (Geisler and Sanders, [9] #239; Geisler et al., [10] #239, [11] #239; modified from Murakami et al. [2, 3] #187; modified from Muizon, [35]; Messenger, [52]; Messenger and McGuire, [8] #1494; Fordyce, [18] #33; Lambert, [7] #55).

(187) Bony connection between posterior process of periotic and squamosal/occipital bones: present (0); absent (ligamentous). (1). (Muizon, [13]; Arnold and Heinsohn,[4] #34; Messenger and McGuire, [8] #1491; Murakami et al. [2, 3] #188; derived from Fraser and Purves, [21]; Kasuya, [51]; Heyning, [22]).

(188) Posterior process of periotic in lateral view: ventrally bent (0); in same plane as body of periotic (1). (Bianucci, [5] #19; Murakami et al. [2, 3] #189; modified from Arnold and Heinsohn,[4] #28; Lambert, [7] #54).

(189*) Angle between posterior process of periotic and long axis of pars cochlearis from dorsal or ventral views: >135° (0); ≤135° (1). (Murakami et al. [2, 3] #190; modified from Geisler and Sanders, [9] #246; Lambert, [7] #54; Geisler et al., [10] #246, [11] #246; derived from Kasuya, [51]; Barnes, [20]; Luo and Marsh, [49]).

(190*) Facet for bulla on posterior process of periotic, parallel-sided; no (0); yes (1). (modified from Fordyce, [18] #63).

(191*) Ventral surface of posterior process of periotic, along a straight path perpendicular to its long axis: flat (0); concave (1); convex (2). (Murakami et al. [2, 3] #191; modified from Geisler and Sanders, [9] #242; Geisler et al., [10] #242, [11] #242).

(192) Posterior bullar facet of periotic: with many long deep grooves and low ridges (0); with some shallow grooves and/or low ridges (1); without grooves or ridges (2). (Bianucci, [5] #20; Murakami et al. [2, 3] #192).

(193) Length of posterior process of periotic as percent length of pars cochlearis: long, ≥85% (0); short, ≤84% (1). (Murakami et al. [2, 3] #193; modified from Barnes, [20]; Luo and Marsh, [49] #24; Geisler and Sanders, [9] #245; Geisler et al., [10] #245, [11] #245).

(194) Mastoid exposure of posterior process of periotic on outside of skull: exposed externally (0); not exposed, enclosed by exoccipital and squamosal (1). (Geisler and Luo, [48] #28; Luo and Marsh, [49] #28; Geisler and Sanders, [9] #249; Geisler et al., [10] #249, [11] #249; Murakami et al. [2, 3] #194).

Tympanic Bulla

(195*) Anterior spine of tympanic bulla: absent (0); present but short (1); present and long (2). (Muizon, [35]; Fordyce, [18] #45; Geisler and Sanders, [9] #250; Lambert, [7] #62; Geisler et al., [10] #250, [11] #250;Murakami et al. [2, 3] #195; modified from Messenger and McGuire, [8] #1484; derived from Kasuya, [51]).

(196) Anterolateral convexity of tympanic bulla with anterolateral notch: absent (0); present (1). (Muizon, [35]; Fordyce, [18] #46; Lambert, [7] #63; Murakami et al. [2, 3]#196).

(197) Articulation of posterior process of tympanic bulla with squamosal: process contacting post-tympanic process of squamosal and posterior process of periotic (0); process contacting periotic only (1). (Muizon, [13]; Fordyce, [18] #29; Arnold and Heinsohn,[4] #34; Messenger and McGuire, [8] #1481; Lambert, [7] #56; Murakami et al. [2, 3] #197; derived Kasuya, [51]).

(198*) Width of tympanic bulla as percentage of its length along its long axis: wide, ≥65% (0); narrow and long, ≤64% (1). (Geisler and Sanders, [9] #251; Bianucci, [5] #23; Geisler et al., [10] #251, [11] #251; Murakami et al. [2, 3] #198; derived from Kasuya, [51]).

(199) Accessory ossicle or homologous region on lip of bulla: not fused (0); fused to anterior process of periotic (1). (Barnes, [20]; Fordyce, [18]; Luo and Marsh, [49]; Geisler and Sanders, [9] #255; Geisler et al., [10] #255, [11] #255; Murakami et al. [2, 3] #199).

(200*) Lateral furrow of tympanic bulla: shallow groove (0); absent (1); deep, well-defined groove (2). (Murakami et al. [2, 3] #200; modified from Muizon, [13, 17]; Arnold and Heinsohn,[4] #31; Messenger and McGuire, [8] #1485; Fajardo-Mellor et al., [15] #17; Lambert, [6] #17; derived from Kasuya, [51]).

(201) Sigmoid process: directed laterally to posterolaterally (0); directed anteriorly to anterolaterally (1). (Murakami et al. [2, 3] #201; modified from Messenger and McGuire, [8] #1486; Lambert, [7] #67, Kasuya, [51]).

(202*) Medial edge of sigmoid process: expanded anteriorly to appose lateral tuberosity of

periotic (0); not articulating with squamosal or periotic (1) (Murakami et al. [2, 3] #202; modified from Geisler and Sanders, [9] #260; Geisler et al., [10] #260, [11] #260; modified from Luo and Marsh, [49] #10).

(203) Ventral margin of tympanic bulla in lateral view: convex (0); concave (1). (Lambert, [7] #66; Murakami et al. [2, 3] #203).

(204) Elliptical foramen of tympanic bulla: present (0); absent or close (1). (Geisler and Sanders, [9] #261; Geisler et al., [10] #261, [11] #261; Murakami et al. [2, 3] #204; derived from Kasuya, [51]).

(205*) Size of posterior process of tympanic bulla: equal to or greater than total length of tympanic bulla (0); much smaller than total length of tympanic bulla (1). (Muizon, [13, 26]; Heyning, [22] #23, 29, [23] #55, 61; Messenger and McGuire, [8] #1482; Murakami et al. [2, 3] #205; modified from Lambert, [7] #57; derived from Yamada, [50]; Kasuya, [51]).

(206) Surface of posterior process of tympanic bulla: spiny or irregular edges (0); cauliflower-like bony growth (1); rounded and pachyostotic (2). (Muizon, [26]; Messenger and McGuire, [8] #1483; Murakami et al. [2, 3] #206; derived from Kasuya, [51]).

(207) Median furrow: short extension on ventral face of interprominental notch (0); anterolateral curvature of median groove to connect to long lateral furrow on outer lip (1); median groove reaching an anterior level beyond lateral furrow, and often slightly curved laterally (2); long and deep rectilinear median groove reaching at least to base of anterior tip of tympanic bulla (3). (Lambert, [7] #64; Murakami et al. [2, 3] #207).

(208) Median furrow on posterior side of bulla: divided by a transverse ridge originating from involucrum (0); transverse ridge absent (1). (Geisler and Sanders, [9] #267; Geisler et al., [10] #267, [11] #267; Murakami et al. [2, 3] #208).

(209) Posterior edge of medial prominence of involucrum: approximately in line with posterior edge of lateral prominence (0); distinctly anterior to posterior edge of lateral prominence (1). (Muizon, [35]; Geisler and Sanders, [9] #269; Geisler et al., [10] #269, [11] #269; Murakami et al. [2, 3] #209; derived from Kasuya, [51]).

(210*) Dorsal margin of involucrum of tympanic bulla: not excavated (0); excavated just anterior to posterior process (1); excavated at mid-part of involucrum (2). (Muizon, [17]; Messenger and McGuire, [8] #1487; Murakami et al. [2, 3] #210, 211; modified from Lambert, [7] #60; Geisler and Sanders, [9] #271; Geisler et al., [10] #271, [11] #271).

(211*) Ridge on inside of bulla: present, as transverse ridge extending laterally from

involucrum and partially dividing cavum tympani into anterior and posterior portions (0); absent (1). (Geisler and Sanders, [9] #272; Geisler et al., [10] #272, [11] #272; Murakami et al. [2, 3] #212).

(212*) Ventromedial keel of tympanic bulla: present along entire length (0); terminating approximately at level of lateral furrow or mid-point of the tympanic bulla (1); poorly defined along entire length (2). (Geisler and Sanders, [9] #273; Geisler et al., [10] #273, [11] #273; Murakami et al. [2, 3] #213; derived from Kasuya, [51]).

(213) Posterior end of ventromedial keel: not protruding and directed medially (0); protruding and directed medially (1). (Geisler and Sanders, [9] #275; Geisler et al., [10] #275, [11] #275; Murakami et al. [2, 3] #214).

Hyals

(214*) Basihyal and thyrohyal connection: unfused (0); fused (1). (Murakami et al. [2, 3] #215; modified from Bianucci, [5] #25).

(215*) Basihyal and thyrohyal shape: arched (0); angled (1). (Murakami et al. [2, 3] #216; modified from Bianucci, [5] #25).

Vertebrae

(216) Dorsal transverse process of atlas: developed dorsolaterally (0); fused with ventral transverse process, with length of process greater than width (1); absent or rudimentary obtuse angle (2). (Murakami et al. [2, 3] #217; modified from Muizon, [17]; Barnes, [20]).

(217) Roof of neural canal of atlas: arched (0); convex (1); straight (2). (Murakami et al. [2, 3] #218).

(218) Postzygapophysis of axis in anterior view: appearing as crest, elongated dorsolaterally (0); appearing as rudimentary crest (1); not appearing (2). (Murakami et al. [2, 3] #219).

(219) Cervical vertebrae: unfused (0); atlas and axis fused (1); C1–C3 or C1–C4 fused (2); C1–C6 or C1–C7 fused (3); C2–C7 fused (4). (Murakami et al. [2, 3] #220; modified from Arnold and Heinsohn,[4] #9; Messenger and McGuire, [8] #1501; Geisler and Sanders, [9] #278, 279; Fajardo-Mellor et al., [15] #18; Lambert, [6] #18; Geisler et al., [10] #278, 279, [11] #278, 279; derived from Allen, [53]; Miller, [28]; Fraser and Noble, [38]; De Smet, [54]; Rommel, [55]).

(220) Length of cervicals (C1–C7) as percent of height of vertebral body plus neural canal of atlas: long, >150% (0); short, <150% (1). (Murakami et al. [2, 3] #221).

(221) Number of thoracic vertebrae: 9–11 (0); 10–12 (1); 13–16 (2); 17–18 (3). (Murakami et al.

[2, 3] #222; modified from Geisler and Sanders, [9] #281; Geisler et al., [10] #281, [11] #281; derived from Sanders and Barnes, [41]).

(222) Capitular articulation facets of posterior vertebrae: facets gradually shift downward on sequential vertebrae to fuse with tubercular facets (0); facets abruptly shift from a position on neural arch to a pedestal, originating from centrum on subsequent vertebra (1). (Geisler and Sanders, [9] #282; Geisler et al., [10] #282, [11] #282; Murakami et al. [2, 3] #223; derived from Flower, [30]; Miller, [28]).

(223) Transverse processes of lumbar vertebrae: extend parallel to anterior and posterior borders (0); triangular (1). (Muizon, [13, 17, 46]; Messenger and McGuire, [8] #1502; Geisler and Sanders, [9] #285; Geisler et al., [10] #285, [11] #285; Murakami et al. [2, 3] #224).

(224) Transverse processes of lumbar vertebrae: oriented ventrolaterally (0); oriented laterally and horizontally (1). (Geisler and Sanders, [9] #284; Geisler et al., [10] #284, [11] #284; Murakami et al. [2, 3] #225; derived from Sanders and Barnes, [41]).

(225) Ratio of greatest breadth of transverse process to width of centrum at anterior face in lumbar vertebrae: some or all lumbar vertebrae >2.5 (0); no lumbar vertebrae >2.5 (1). (Murakami et al. [2, 3] #226).

(226) Number of lumbar vertebrae: 6–8 (0); 9–12 (1); 13–15 (2); 16–19 (3); 20–25 (4); >26 (5). (Murakami et al. [2, 3] #227; modified from Geisler and Sanders, [9] #287, [11] #287; Bianucci, [5] #39; Geisler et al., [10] #287).

(227) Number of caudal vertebrae: 15–20 (0); 21–27 (1); 28–33 (2); 34–40 (3); >41 (4). (Murakami et al. [2, 3] #228; modified from Geisler and Sanders, [9] #288; Bianucci, [5] #40; Geisler et al., [10] #288, [11] #288).

Sternum and Sternal Ribs

(228) Sternum: consists of four or five parts (0); consists of two or three parts (1); consists of single bone (2). (Murakami et al. [2, 3] #229; modified from Geisler and Sanders, [9] #290; Geisler et al., [10] #290; derived from Yablokov, [56]; Van Valen, [57]).

(229) Ventrolateral processes on manubrium of sternum: absent (0); present but small, occur ventral to articulation surface of first costal cartilage or rib (1). (Muizon, [17]; Messenger and McGuire, [8] #1503; Geisler and Sanders, [9] #289; Geisler et al., [10] #289, [11] #289; Murakami et al. [2, 3] #230; derived from Klima et al., [58]).

(230) Sternal ribs: unossified or ossification of fewer than five pairs (0); ossification of five pairs or more (1). (Murakami et al. [2, 3] #231; derived from Flower, [37]).

(231) Number of two head ribs: ≥9(0); 8 (1); ≤7 (2). (Murakami et al. [3] #282).

Scapula

(232) Anterodorsal part of scapula: rounded (0); rounded and anterior edge pointed (1); almost rectilinear (2). (Murakami et al. [2, 3] #232).

(233) Ventral projection on anterior border of scapula: absent (0); present (1). (Fajardo-Mellor et al., [15] #26; Murakami et al. [2, 3] #233; derived from Noble and Fraser, [38]).

(234*) Anterior slope on scapula between anterior angle and midpoint of glenoid fossa with anterior and posterior margin of glenoid fossa on a plane: shallow, <35° (0); steeper, >35° (1). (modified from Murakami et al. [2, 3] #234; modified from Bianucci, [5] #31).

(235*) Posterior slope on scapula, between scapula and midpoint of glenoid fossa with anterior and posterior margin of glenoid fossa on a plane: shallow, <25° (0); steeper, >25° (1). (modified from Murakami et al. [2, 3] #235; modified from Bianucci, [5] #32).

(236*) Crest between infraspinous fossa and teres fossa: weakly developed (0); strongly developed (1). (Murakami et al. [2, 3] #236).

(237) Coracoid process of scapula: not expanded distally (0); expanded distally (1); notably reduced or absent (2). (Murakami et al. [2, 3] #237; modified from Muizon, [34, 35]; Messenger and McGuire, [8] #1504; Geisler and Sanders, [9] #292; Lambert, [7] #73; Bianucci, [5] #33; Geisler et al., [10] #292; derived from True, [59]).

(238) Coracoid process of scapula, with glenoid fossa directed ventrally: directed horizontally (0); directed nearly anterodorsally (1); directed anteroventrally (2). (Murakami et al. [2, 3] #238; modified Barnes, [20]).

(239) Acromion of scapula: narrow and not expanded distally (0); expanded distally (1). (Murakami et al. [2, 3] #239; modified from Bianucci, [5] #34).

(240) Acromion of scapula, when glenoid fossa direct ventrally: directed horizontally (0); directed anterodorsally (1); directed anteroventrally (2). (Murakami et al. [2, 3] #240; modified from Barnes, [20]).

(241) Supraspinous fossa of scapula: present (0); absent or nearly absent (1). (Muizon, [34, 35]; Geisler and Sanders, [9] #293; Lambert, [7] #72; Geisler et al., [10, 11] #293; Murakami et al. [2, 3] #241).

Forelimb (except scapula)

(242) Ratio of length of humerus to length of radius: long, >1.1 (0); short, <0.8 (1). (Murakami et al. [2, 3] #242; modified from Sanders and Barnes, [41]; Geisler and Sanders, [9] #297; Geisler et al., [10, 11] #297).

(243) Location of apex of deltopectoral tuberosity of humerus: within proximal 65% of humerus (0); within distal 35% of humerus (1). (Murakami et al. [2, 3] #243; modified from Muizon, [17]; Messenger and McGuire, [8] #1506; Geisler and Sanders, [9] #295; Bianucci, [5] #35; Geisler et al., [10] #295, [11] #295).

(244) Prominent deltoid crest on anterior edge of humerus: present, forms greatest anteroposterior diameter along shaft (0); forming a knob-like tuberosity (1); neither tuberosity or crest absent (2). (Geisler and Sanders, [9] #294; Geisler et al., [10] #294, [11] #294; Murakami et al. [2, 3] #244; derived from Sanders and Barnes, [41]).

(245) Radial and ulnar facets of humerus in lateral view: facets forming a semicircular articulation surface (0); facets forming an obtuse angle (1). (Barnes, [20]; Geisler and Sanders, [9] #296; Geisler et al., [10] #296, [11] #296; Murakami et al. [2, 3] #245).

(246) Olecranon process: present as a distinct process (0); present as a slightly raised proximal posterior edge (1); absent (2). (Messenger and McGuire, [8] #1507; Geisler and Sanders, [9] #296; Geisler et al., [10] #284, [11] #284; Murakami et al. [2, 3] #246; modified from Muizon, [13]; Barnes, [20]; Arnold and Heinsohn,[4] #10; Fajardo-Mellor et al., [15] #28; derived from Howell, [60]).

Soft Tissues

(247) Spermaceti organ: absent (0); present (1). (Fordyce, [18] #17; Messenger and McGuire, [8] #1511; Geisler and Sanders, [9] #97; Geisler et al., [10] #97, [11] #97; Murakami et al. [2, 3] #247; derived from Norris and Harvey, [61]; Cranford et al., [62]).

(248) Museau de singe: absent (0); present (1). (Messenger and McGuire, [8] #1512; Murakami et al. [2, 3] #248; derived from Norris, [63]; Cranford et al., [62]).

(249) Lateral lips of nasal plug: present (0); absent (1). (Messenger and McGuire, [8] #1523; Murakami et al. [2, 3] #249).

(250) Proximal sac: single frontal sac (0); sac complex, with nasofrontal sacs and vestibule (1). (Heyning, [22] #6, 11, 17, [23] #33, 43, 49; Fordyce, [18] #16; Messenger and McGuire, [8] #1531, 1532; Lambert, [7] #18; Murakami et al. [2, 3] #250).

(251) Posterior nasal sacs: absent (0); present (1). (Heyning, [22] #41, [23] #73; Arnold and Heinsohn,[4] #6; Messenger and McGuire, [8] #1534; Murakami et al. [2, 3] #251).

(252) Posterior nasal sacs: single (0); divided (1). (Messenger and McGuire, [8] #1535; Murakami et al. [2] #252).

(253) Anterior section of nasofrontal sac: absent (0); present (1). (Messenger and McGuire, [8] #1536; Murakami et al. [2, 3] #253).

(254) Anterior part of nasofrontal sac: smooth (0); trabeculate (1). (Messenger and McGuire, [8] #1537; Fajardo-Mellor et al., [15] #35; Murakami et al. [2, 3] #254; derived from Heyning, [22]).

(255) Vestibular sac: absent (0); present (1); hypertrophied (2). (Heyning, [22] #28, [23] #60; Fordyce, [18] #31; Arnold and Heinsohn,[4] #1, 3; Messenger and McGuire, [8] #1541; Lambert, [7] #17; Fajardo-Mellor et al., [15] #39; Murakami et al. [2, 3] #255).

(256) Floor of vestibular sac (nasal sac): not rigid (0); rigid (1). (Heyning, [22] #38, [23] #70; Arnold and Heinsohn,[4] #2; Messenger and McGuire, [8] #1543; Fajardo-Mellor et al., [15] #38; Murakami et al. [2, 3] #256).

(257) Vestibular sac (nasal sac): undivided (0); bilaterally divided (1). (Messenger and McGuire, [8] #1544; Fajardo-Mellor et al., [15] #36; Murakami et al. [2, 3] #257; derived from Heyning, [22]).

(258) Right and left sides of vestibular sac (nasal sac): same size (0); right side larger than left (1). (Heyning, [22] #30, [23] #62; Messenger and McGuire, [8] #1545; Murakami et al. [2, 3] #258).

(259) Intrinsic muscle in vestibular sac (nasal sac): absent (0); present (1). (Messenger and McGuire, [8] #1546; Fajardo-Mellor et al., [15] #37; Murakami et al. [2, 3] #259; derived from Mead, [64]).

(260) Floor of vestibular sac (nasal sac): smooth (0); wrinkled (1). (Heyning, [23] #70; Arnold and Heinsohn,[4] #2; Messenger and McGuire, [8] #1543; Murakami et al. [2, 3] #260).

(261) Diagonal membrane: absent (0); present (1). (Messenger and McGuire, [8] #1550; Murakami et al. [2, 3] #261; Heyning, [22]).

(262) Spiracular cavity: slit-like (0); rounded (1). (Messenger and McGuire, [8] #1552; Murakami et al. [2, 3] #262).

(263) Pars posteroexternus muscle: absent (0); present (1). (Messenger and McGuire, [8] #1553; Murakami et al. [2, 3] #263).

(264) Pars intermedius muscle: absent (0); present (1). (Messenger and McGuire, [8] #1554; Murakami et al. [2, 3] #264).

(265) Pars posterointerus muscle: absent (0); present (1). (Messenger and McGuire, [8] #1556; Murakami et al. [2, 3] #265).

(266) Pars anterointerus muscle: one insertion (0); two insertions (1). (Messenger and McGuire, [8] #1557; Murakami et al. [2, 3] #266).

(267) Blowhole shape: longitudinal slit, may be slightly sigmoidal or angled (0); crescent, with

apices pointed anteriorly (1); crescent, with apices pointed posteriorly, may be skewed (2); rectangular (3). (Murakami et al. [2, 3] #267; modified from Messenger and McGuire, [8] #1525).

(268) Soft tissues of nasal passages distal to bony external nares: separated for most of their length but confluent just proximal to blowhole (0); confluent (1). (Heyning, [22]; Fordyce, [18] #20; Messenger and McGuire, [8] #1529; Geisler and Sanders, [9] #95; Lambert, [7] #16; Geisler et al., [10] #95, [11] #95; Murakami et al. [2, 3] #268).

(269) Distal sac: absent (0); present, situated immediately distal to museau de singe (1). (Murakami et al. [2, 3] #269; modified from Heyning, [22] #12, [23]#44; Fordyce, [18] #14; Messenger and McGuire, [8] #1533; Geisler and Sanders, [9] #99, [11] #99; Lambert, [7] #19; Geisler et al., [10] #99).

(270) Blowhole ligament: absent (0); present (1). (Heyning, [22] #15, [23] #44; Fordyce, [18] #13; Messenger and McGuire, [8] #1527; Geisler and Sanders, [9] #101; Lambert, [7] #20; Geisler et al., [10] #101, [11] #101; Murakami et al. [2, 3] #270).

(271) Blowhole ligament: not appressed against skull (0); appressed against skull (1). (Messenger and McGuire, [8] #1528; Murakami et al. [2, 3] #271).

(272) Cartilage on blowhole ligament: absent (0); present (1). (Messenger and McGuire, [8] #1529; Murakami et al. [2, 3] #272).

(273) Premaxillary sac: absent (0); present (1). (Heyning, [22] #16, [23], #48; Messenger and McGuire, [8] #1538; Geisler and Sanders, [9] #105; Geisler et al., [10] #105, [11] #105; Murakami et al. [2, 3] #273).

(274) Accessory sac: absent (0); present, forms small diverticulum of inferior vestibule and extends anterolaterally around the attachment of blowhole ligament to the premaxilla (1). (Messenger and McGuire, [8] #1549; Geisler and Sanders, [9] #106; Fajardo-Mellor et al., [15] #40; Geisler et al., [10] #106, [11] #106; Murakami et al. [2, 3] #274; derived from Schenkkan, [65]; Mead, [64]; Heyning, [22]).

(275) Esophageal forestomach: present (0); absent (1). (Geisler and Sanders, [9] #300; Geisler et al., [10] #300, [11] #300; Murakami et al. [2, 3] #275; derived from Mead, [66]; Rice and Wolman, [67]).

(276) External throat grooves: absent (0); one pair converged anteriorly (1); irregular in number and shape (2). (Murakami et al. [2] #276; modified from Messenger and McGuire, [8] #1512, 1513; Geisler and Sanders, [9] #301; Geisler et al., [10] #301, [11] #301).

(277) Dorsal fin: present (0); dorsal hump (1); absent (2). (Murakami et al. [2] #277; modified from Messenger and McGuire, [8] #1562; Geisler and Sanders, [9] #304; Geisler and Sanders, 2003 #304, [11] #304; derived from Leatherwood and Reeves, [68]; Jefferson and Newcomer [69], Reeves et al. [70]).

(278) Shape of flipper: fan shaped (0); rounded at tip (1); sharply pointed at tip (2); entire flipper rounded (3). (Murakami et al. [2] #278; modified from Fajardo-Mellor et al., [15] #31; Lambert, [6] #25; derived from Leatherwood and Reeves [68], Brownell et al. [71]).

(279) Intra-premaxillary foramen on posterior dorsal surface of skull, which is bounded by premaxilla and maxilla: absent (0); present (1).

(280) Shape of restricted area between postorbital ridge of frontal and subtemporal crest from ventral view: anteroposteriorly long elliptical (0); wide fan-shape (1); narrow fan-shape (2), rhombus (3).

(281) Temporal fossa shape in lateral view: height lower than anteroposterior length (0); higher (1); nearly equilateral square (2); lower and its posterior end is rounded (3).

(282) Anterior zygomatic process end of squmosal in lateral view: taipered (0); squared (1).

(283) Basioccipital width compared with maximum width of skull in ventral view: narrow, less than 50% (0), wider larger than 51% (1).

(284) Occipital condyles; on pedicle (0); lacking pedicle, unified with occipital (1).

(285) Incisors relatively delicate and procumbent: no (0); yes (1). (Fordyce (1994) #42).

(286) Crown of heterodont teeth: long (>10 mm) (0); short (<10mm) (1). (Fordyce (1994) #58).

(287) Large incisor tusk: absent (0); present (1).

(288~) Profile of anterior process of periotic ventrally deflected in lateral view: no, has crudely rectangular profile (0); smoothly deflected (1); abruptly deflected (2). (Fordyce (1994) #25).

(289~) Cochlear aqueduct on periotic large with a thin edge: no (0); yes (1). (Fordyce (1994) #28).

(290~) Thickness dorsoventral of pars cochlearis on periotic; thick (0) flat (1) (Gutstein et al. (2014)).

(291~) Profie of cochlear on periotic in dorsoventral; rounded (0), sub-rectangular (1), squared (2). (modified from Fordyce (1994) #61; Bianucci et al. (2013) #2).

(292) Acromion process of scapula lies on anterior edge, with loss of supraspinous fossa: no (0); yes (1). (de Muizon (1987); Fordyce (1994) #48).

Coding additions by Boersma & Pyenson (2016):

(288)

Pomatodelphis [0]

Zarhachis [0]

(289)

Pomatodelphis [0]

Zarhachis [0]

(290)

Pomatodelphis [1]

Zarhachis [1]

(291)

Pomatodelphis [2]

Zarhachis [2]

References

1. Mead JG and Fordyce RE (2009) The therian skull: a lexicon with emphasis on the odontocetes. Smithson Contrib Zool 627: 1-248.
2. Murakami M, Shimada C, Hikida Y and Hirano H (2012) A new basal Porpoise, Pterophocaena nishinoi (Cetacea, Odontoceti, Delphinoidea), from the upper Miocene of Japan and its phylogenetic relationships. J Vertebr Paleontol 32: 1157-1171.
3. Murakami M, Shimada C, Hikida Y and Hirano H (2012) Two new extinct basal phocoenids (Cetacea, Odontoceti, Delphinoidea), from the upper Miocene Koetoi Formation of Japan and their phylogenetic significance. J Vertebr Paleontol 32: 1172-1185.
4. Arnold PW and Heinsohn GE (1996) Phylogenetic status of the Irrawaddy dolphin Orcaella brevirostris (Owen in Gray): a cladistic analysis. Mem Queensland Mus 39: 141-204.
5. Bianucci G (2005) Arimidelphis sorbinii a new small killer whale-like dolphin from the Pliocene of Marecchia River (Central eastern Italy) and a phylogenetic analysis of the Orcininae (Cetacea: Odontoceti). Riv Ital Paleontol Stratigr 111: 329-344.
6. Lambert O (2008) A new porpoise (Cetacea, Odontoceti, Phocoenidae) from the Pliocene of the North Sea. J Vertebr Paleontol 28: 863-872.
7. Lambert O (2005) Phylogenetic affinities of the long-snouted dolphin Eurhinodelphis (Cetacea, Odontoceti) from the Miocene of Antwerp, Belgium. Palaeontology 48: 653-679.
8. Messenger SL and McGuire JA (1998) Morphology, molecules, and the phylogenetics of cetaceans. Syst Biol 47: 90-124.
9. Geisler JH and Sanders AE (2003) Morphological evidence for the phylogeny of Cetacea. J Mamm Evol 10: 23-129.
10. Geisler JH, McGowen MR, Yang G and Gatesy J (2011) A supermatrix analysis of genomic, morphological, and paleontological data from crown Cetacea. BMC Evol Biol 11: 112.

11. Geisler JH, Godfrey SJ and Lambert O (2012) A new genus and species of late Miocene inioid (Cetacea, Odontoceti) from the Meherrin River, North Carolina, USA. J Vertebr Paleontol 32: 198-211.
12. Moore JC (1968) Relationships among the living genera of beaked whales with classifications, diagnoses and keys. Fieldiana, Zool 53: 509-598.
13. de Muizon C (1984) Les vertébrés fossiles de la Formation Pisco (Pérou). deuxiéme partie: les Odontocétes (Cetacea, Mammalia) du Pliocéne inférieur de Sud-Sacaco. Trav Inst Fr Et Andines 27: 1-188.
14. Barnes LG (1985) Fossil pontoporiid dolphins (Mammalia: Cetacea) from the Pacific coast of North America. Contributions to Science, Natural History Museum of Los Angeles County 363: 1-34.
15. Fajardo-Mellor L, Berta A, Brownell RL, Boy CC and Goodall NP (2006) The phylogenetic relationships and biogeography of true porpoises (Mammalia: Phocoenidae) based on morphological data. Mar Mamm Sci 22: 910-932.
16. Aguirre-Fernández G, Barnes LG, Aranda-Manteca FJ and Fernández-Rivera JR (2009) Protoglobicephala mexicana, a new genus and species of Pliocene fossil dolphin (Cetacea; Odontoceti; Delphinidae) from the Gulf of California, Mexico. Bol Soc Geo Mex 61: 245-265.
17. de Muizon C (1988) Les relations phylogenetiques des Delphinida (Cetacea, mammalia). Ann Paleontol 74: 159-227.
18. Fordyce RE (1994) Waipatia maerewhenua, new genus and new species (Waipatiidae, new family), an archaic Late Oligocene dolphin (Cetacea: Odontoceti: Platanistoidea) from New Zealand. Proc San Diego Soc Nat Hist 29: 147-176.
19. Zhou K (1982) Classification and phylogeny of the superfamily Platanistoidea, with notes on evidence of the monophyly of the Cetacea. Sci Rep Whales Res Inst Tokyo: 93-108.
20. Barnes LG (1990) The fossil record and evolutionary relationships of the genus Tursiops. In: S. Leatherwood and R. R. Reeves, editors. The bottlenose dolphin. San Diego, New York: Academic Press Inc. pp. 3-26.
21. Fraser FC and Purves PE (1960) Hearing in cetaceans: evolution of the accessory air sacs and the structure of the outer and middle ear in recent cetaceans. Bull BMNH zool 7: 1-140.
22. Heyning JE (1989) Comparative facial anatomy of beaked whales (Ziphiidae) and a systematic revision among the families of extant Odontoceti. Contrib Sci, Nat Hist Mus LAC 405: 1-64.
23. Heyning JE (1997) Sperm whale phylogeny revisited: analysis of the morphological evidence. Mar Mamm Sci 13: 596-613.
24. Barnes LG (1984) Fossil odontocetes (Mammalia: Cetacea) from the Almejas Formation, Isla Cedros, Mexico. Museum of Paleontology, University of California.
25. Flower WH (1872) On the recent ziphioid whales, with a description of the skeleton of Berardius arnouxi. The Transactions of the Zoological Society of London 8: 203-234.
26. de Muizon C (1991) A new Ziphiidae (Cetacea) from the Early Miocene of Washington State (USA) and phylogenetic analysis of the major groups of odontocetes. B Mus Natl Hist Nat 12: 279-326.
27. Flower WH (1885) List of the specimens of Cetacea in the Zoological Department of the British Museum. London: British Museum. 36 p.
28. Miller GS (1923) The telescoping of the cetacean skull. Smiths misc coll 76: 1-70.
29. Kellogg R (1923) Description of an Apparently New Toothed Cetacean from South Carolina:(with Two Plates). Smiths Misc Coll 76: 1-7.
30. Flower WH (1868) On the osteology of the cachalot or sperm-whale (Physeter

macrocephalus). Trans Zool Soc Lond 6: 309-372.
31. Schulte HvW (1917) The skull of Kogia breviceps Blainv. Bull Am Mus Nat Hist: 361-404.
32. Barnes LG (1984) Whales, dolphins and porpoises; origin and evolution of the Cetacea. In: P. D. Gingerich and C. E. Badgle, editors. Mammals Notes for a short course. University of ATennessee, Department of Geological Science.
33. Barnes LG (1985) Evolution, taxonomy and antitropical distributions of the porpoises (Phocoenidae, Mammalia). Mar Mamm Sci 1: 149-165.
34. de Muizon C (1994) Are the squalodonts related to the platanistoids? Proc San Diego Soc Nat Hist 29: 135-146.
35. de Muizon C (1987) The affinities of Notocetus vanbenedeni, an early Miocene platanistoid (Cetacea, Mammalia) from Patagonia, Southern Argentina. Am Mus Novit 2904: 1-20.
36. Curry BE (1992) Facial anatomy and potential function of facial structures for sound production in the harbor porpoise (Phocoena phocoena) and Dall's porpoise (Phocoenoides dalli). Can J Zool 70: 2103-2114.
37. Flower WH (1867) Description of the skeleton of Inia geoffrensis and the skull of Pontoporia blainvillii, with remarks on the systematic position of these animals in the Order Cetacea. Trans Zool Soc Lond 6: 87-116.
38. Noble B and Fraser F (1971) Description of a skeleton and supplementary notes on the skull of a rare porpoise Phocoena sinus Norris & McFarland 1958. J Nat Hist 5: 447-464.
39. Kuzmin AA (1976) Embryogenesis of the osseous skull of the sperm whale (Physeter macrocephalus Linnaeus, 1758). Invest Cetacea 7: 187-202.
40. Whitmore FC and Sanders AE (1977) Review of the Oligocene Cetacea. Syst Zool 25: 304-320.
41. Sanders AE and Barnes LG (2002) Paleontology of the late Oligocene Ashley and Chandler Bridge formations of South Carolina, 2: Micromysticetus rothauseni, a primitive cetotheriid mysticete (Mammalia: Cetacea). Smithson Contrib Paleobiol: 271-293.
42. Flower WH (1884) On the characters and divisions of the Family Delphinidae. Proc Zool Soc Lond 1883: 466-513.
43. Marsh H, Lloze R, Heinsohn GE and Kasuya T (1989) Irrawaddy dolphin - Orcaella brevirostris, (Gray, 1866). In: S. H. Ridgway and S. Harrison, editors. Handbook of marine mammals Volume 4: river dolphins and the larger toothed whales. pp. 101-118.
44. Kellogg R (1936) A review of the Archaeoceti. Carnegie Institution of Washington publication 482: 1-366.
45. Fordyce RE (2002) Simocetus rayi (Odontoceti: Simocetidae, new family): A bizarre new archaic Oligocene dolphin from the eastern North Pacific. Smithson Contrib Paleobiol 93: 185-222.
46. Muizon Cd (1985) Nouvelles données sur le diphylétisme des Dauphins de rivière (Odontoceti, Cetacea, Mammalia). Comptes rendus l'Academie des Sciences series 2, 301: 359-362.
47. Doran AH (1878) Morphology of the mammalian ossicula auditus. Trans Linn Soc, ser 2, zool 1: 371-497.
48. Geisler JH and Luo Z (1996) The petrosal and inner ear of Herpetocetus sp.(Mammalia: Cetacea) and their implications for the phylogeny and hearing of archaic mysticetes. J Paleontol: 1045-1066.
49. Luo Z and Marsh K (1996) Petrosal (periotic) and inner ear of a Pliocene kogiine whale

(Kogiinae, Odontoceti): implications on relationships and hearing evolution of toothed whales. J Vertebr Paleontol 16: 328-348.
50. Yamada M (1953) Contribution to the anatomy of the organ of hearing of whales. Sci Rep Whales Res Inst 8: 1-79.
51. Kasuya T (1973) Systematic consideration of recent toothed whales based on the morphology of tympano-periotic bone. Sci Rep Whales Res Inst 25: 1-103.
52. Messenger S (1994) Phylogenetic relationships of platanistoid river dolphins (Odontoceti, Cetacea): assessing the significance of fossil taxa. Contributions in Marine Mammal Paleontology Honoring Frank Whitmore Jr Proc San Diego Soc Nat Hist: 125-133.
53. Allen GM (1923) The black finless porpoise, Meomeris. Bull Mus Comp Zool 65: 233-256.
54. De Smet WMA (1977) The regions of the cetacean vertebral column volume 3. In: R. J. Harrison, editor editors. Functional anatomy of marine mammals. London: Academic Press. pp. 59-80.
55. Rommel S (1990) Osteology of the bottlenose dolphin. In: S. Leatherwood and R. R. Reeves, editors. The bottlenose dolphin. San Diego, New York: Academic Press Inc. pp. 29-49.
56. Yablokov A (1964) Convergence or parallelism in the evolution of cetaceans. Int Geol Rev 7: 1461-1468.
57. Van Valen L (1968) Monophyly or diphyly in the origin of whales. Evolution: 37-41.
58. Klima M, Oelschlaeger HA and Wünsch D (1980) Morphology of the pectoral girdle in the Amazon dolphin Inia geoffrensis with special reference to the shoulder joint and the movements of the flippers. Zeitschrift Saugertierkunde 45: 288-309.
59. True FW (1904) The whalebone whales of the western North Atlantic compared with those occurring in European waters with some observations on the species of the North Pacific. Smithson contrib knowl 33: 1-332.
60. Howell AB (1927) Contribution to the anatomy of the Chinese finless porpoise Neomeris phocaenoides. Proc US Natl Mus 70: 1-43.
61. Norris KS and Harvey GW (1972) A theory for the function of the spermaceti organ of the sperm whale (Physeter catodon L). NASA Special Publication 262: 397.
62. Cranford TW, Amundin M and Norris KS (1996) Functional morphology and homology in the odontocete nasal complex: implications for sound generation. J Morphol 228: 223-285.
63. Norris KS (1964) Some problems of echolocation in cetaceans. In: W. N. Tavolga, editor editors. Marine bio-acoustics. New York: MacMillan. pp. 317-336.
64. Mead JG (1975) Anatomy of the external nasal passages and facial complex in the Delphinidae(Mammalia, Cetacea). Smiths contrib zool 207: 1-72.
65. Schenkkan E (1971) The occurrence and position of the “connecting sac” in the nasal tract complex of small odontocetes (Mammalia, Cetacea). Beaufortia 19: 37-43.
66. Mead J (1989) Shepherd’s beaked whale Tasmacetus shepherdi Oliver, 1937. Handbook of marine mammals 4: 309-320.
67. Rice DW and Wolman AA (1990) The stomach of Kogia breviceps. J Mammal: 237-242.
68. Leatherwood S, Reeves RR and Foster L (1983) Sierra Club handbook of whales and dolphins. San Francisco: Sierra Club Books.
69. Jefferson TA and Newcomer MW (1993) Lissodelphis borealis. Mamm Species: 1-6.
70. Reeves R, Stewart B, Clapham P and Powell J (2002) Marine mammals of the world. Chanticleer Press, Inc, New York.
71. Brownell RL, Findley LT, Vidal O, Robles A and Silvia Manzanilla N (1987) External

morphology and pigmentation of the vaquita, Phocoena sinus (Cetacea: Mammalia). Mar Mamm Sci 3: 22-30.
